# Semiflexible Chains at Surfaces: Worm-Like Chains and beyond

**DOI:** 10.3390/polym8080286

**Published:** 2016-08-08

**Authors:** Jörg Baschnagel, Hendrik Meyer, Joachim Wittmer, Igor Kulić, Hervé Mohrbach, Falko Ziebert, Gi-Moon Nam, Nam-Kyung Lee, Albert Johner

**Affiliations:** 1 Institut Charles Sadron, CNRS-UdS, 23 rue du Loess, BP 84047, 67034 Strasbourg cedex 2, France; jorg.baschnagel@ics-cnrs.unistra.fr (J.B.); hendrik.meyer@ics-cnrs.unistra.fr (H.M.); joachim.wittmer@ics-cnrs.unistra.fr (J.W.); kulic@unistra.fr (I.K.); herve.mohrbach@univ-lorraine.fr (H.M.); fziebert@gmail.com (F.Z.); namg@purdue.edu (G.-M.N.); lee_namkyung@yahoo.com (N.-K.L.); 2 Equipe BioPhysStat Université de Lorraine, 1 boulevard Arago, 57070 Metz, France; 3 Physikalisches Institut, Albert-Ludwigs-Universität Freiburg, Hermann-Herder-Strasse 3, 79104 Freiburg, Germany; 4 Department of Physics, Sejong University, Neundongro 209, Seoul 05006, Korea

**Keywords:** semiflexible polymers, polymers at interfaces, biopolymers

## Abstract

We give an extended review of recent numerical and analytical studies on semiflexible chains near surfaces undertaken at Institut Charles Sadron (sometimes in collaboration) with a focus on static properties. The statistical physics of thin confined layers, strict two-dimensional (2D) layers and adsorption layers (both at equilibrium with the dilute bath and from irreversible chemisorption) are discussed for the well-known worm-like-chain (WLC) model. There is mounting evidence that biofilaments (except stable d-DNA) are not fully described by the WLC model. A number of augmented models, like the (super) helical WLC model, the polymorphic model of microtubules (MT) and a model with (strongly) nonlinear flexural elasticity are presented, and some aspects of their surface behavior are analyzed. In many cases, we use approaches different from those in our previous work, give additional results and try to adopt a more general point of view with the hope to shed some light on this complex field.

## 1. Introduction

Polymers are large molecules comprising one or a few repeating chemical motives called monomers. Classically, monomers are linked by chemical bonds, but polymers can also consist of associated surfactants or proteins. They are widespread in nature and industry. Most technical applications use the unique rheological properties of polymer solutions or melts [1,2]. In nature, specific proteins can associate into biofilaments [3]. Microtubules (MT) associated from tubulins serve as beams, tracks for trafficking [3,4] or stirring rods [5] inside the cell. Proteins like G-actin build filaments (F-actin) that can further associate with molecular motors into active gels [6].

Polymer backbones can be considered as slender objects, which can be described by a few geometrical parameters, like preferred curvature and twist, and mechanical parameters, like their bending and twist modulus. In some instances, the detailed microscopic mechanism of flexibility [7] nonetheless matters. Most polymers possess a (possibly weak) bending modulus *B*. Being neither perfectly flexible, nor perfectly stiff rods, they can be termed semiflexible. One way to quantify flexibility is to evaluate thermal bending fluctuations, which merely depend on the persistence length $\ell =B/(k_{B}T)$. If the contour length *S* of the polymer chain is much larger than *ℓ*, the polymer is essentially flexible at a large scale, albeit fragments (chain sections) shorter than *ℓ* appear stiff (rod-like). Chains with a persistence length *ℓ* comparable to the monomer size *b* appear flexible in most instances. Sometimes, the rigidity is measured by the aspect ratio ℓ/b of the persistent segment. Several examples are listed in Table 1.

Models for ideal polymer chains, which neglect monomer/monomer interactions and only retain chain connectivity, were devised early [7]. The simplest model neglects any type of correlation between neighboring monomers and represents a configuration as a realization of a random walk (RW). It is a reasonable model for a fully-flexible chain without interactions and, to a first approximation, for melts and dense solutions where interactions between monomers are screened. The possibility for two neighboring monomers to overlap or for the RW to allow for immediate return is not realistic. This is cured in a model with fixed bond-angle entailing orientation correlations between neighboring bonds, but still ignoring interactions. This model has a continuous counterpart called the worm-like-chain (WLC) model, which penalizes curvature away from the straight state. Similarly, twist rigidity can be introduced to complement the WLC model.

Ideal chain models generically fail qualitatively for very long chains dissolved in good solvents, which are swollen by excluded volume repulsions. For simplicity, let us consider a chain of spherical monomers of diameter *b* each. For RW configurations comprising *N* steps of step size *b* the internal concentration $∼N/{\left(Nb^{2}\right)}^{D/2}$ with *D* the dimension of space leads to an average number of contacts distant along the chain $∼N^{2}/N^{D/2}$, diverging with *N* for D<4. Let us detail the interaction a bit further in 3D and 2D. In a virial expansion, the interaction associated with the second viral $a_{2}$ reads $a_{2}b^{-D}N^{2-D/2}$. Let us introduce the dimensionless excluded volume $a_{2}/b^{D}$, which is of order one in good solvent. Unless the solvent is only marginal ($a_{2}\ll b^{D}$), even a short chain experiences an interaction energy larger than the thermal energy $k_{B}T$ and is swollen; the ideal RW configurations are not relevant.

The interaction is obviously less important for somewhat stiffer chains, which are less likely to fold back and self-interact. At large scales, the long WLC can still be qualitatively seen as an RW composed of $\tilde{N}∼Nb/\ell$ straight steps of length ∼ℓ with an overall geometrical size $R∼\sqrt{\tilde{N}}\ell$. The interaction becomes $a_{2}{\tilde{N}}^{2}/R^{D}$. The second virial increases with the rigidity as $a_{2}∼\ell^{2}b$ in 3D and $a_{2}∼\ell^{2}$ in 2D. Nonetheless, in 3D, the overall self-interaction inside the coil scales as $\sqrt{N{\left(b/\ell \right)}^{3}}$ and strongly decreases with rigidity. In 3D, chains are almost ideal up to $∼{\left(\ell /b\right)}^{3}$ monomers, which may be less than 10 monomers for some synthetic polymers, but is very large for stiff biopolymers like d-DNA. In 2D, the interaction ∼Nb/ℓ is less affected by rigidity, and the excluded volume matters as soon as the chain becomes “flexible” Nb>ℓ. In summary, in 3D, there is a regime $\ell /b<N<{\left(\ell /b\right)}^{3}$, which is pretty extended for rather stiff chains, where chains are flexible and ideal. The WLC model has some practical relevance in 3D. Such a regime barely exists in 2D as also documented in simulations by Hsu, Paul and Binder [8]. The flexible regime of the WLC model is hence hardly pertinent to the isolated chain in 2D. However, the WLC model can describe the weakly fluctuating chain of contour length S≲ℓ.

Let us now consider an isotropic concentrated solution of semiflexible chains at monomer concentration *c*. In the mean-field, the interaction energy per unit volume reads $a_{2}{\tilde{c}}^{2}$ with $\tilde{c}∼cb/\ell$ the concentration in persistent segments of length *ℓ*. The persistence length cancels out in the interaction energy density. For a high enough concentration, nematic order is anticipated, and the isotropic description is expected to fail. This happens when the correlation length *ξ* of concentration fluctuations is shorter than the persistence length *ℓ*. For stiff chains, the isotropic description is more pertinent to concentrated solution than to strict melts. Away from this (important) transition, rigidity is hence less important in concentrated solutions than for isolated coils in 3D. Long ago, it was proposed by Flory [9] that in dense solutions, or in melts, the excluded volume is screened and the polymer configurations remain ideal. This encourages us to use mean-field theory to describe these systems. Recently, we have shown theoretically and also by simulations for rather flexible chains that screening is not complete and that the tangent/tangent correlation function shows a power law decay at large distances along the chain [10,11]. We will come back to this below.

Polyelectrolytes are an important class of water-soluble polymers. Polyelectrolytes are electrically-charged along their backbone and are accompanied by oppositely-charged counterions. The energetic ground state corresponds to counterions condensed onto the backbone charges, which ensures that the electrical field vanishes everywhere. Unless the electrostatic interaction is very strong as in media with low dielectric constants, the counterions are (at least partially) dispersed in the solvent, say water, to optimize the free energy. The solution also typically contains salt, which entails Debye screening of the electrostatic interaction. In the simplest model, polyelectrolytes are seen as polymers whose monomers interact via the 3D screened electrostatic Yukawa-like potential $q^{2}\frac{l_{B}}{r}e^{-\kappa_{D}r}$ expressed in thermal units between charges *q* counted in elementary charges with $l_{B}$ the Bjerrum length (≈0.7 nm in water) and $\kappa_{D}$ the inverse Debye screening length. Upon bending of the polymer, the energy associated with the screened potential increases, and there is so-called electrostatic stiffening. Electrostatic stiffening turned out to be a delicate matter. Beyond the case of an intrinsically stiff chain, additionally stiffened by electrostatics (say as a perturbation) where the electrostatic contribution to the persistence length is inversely proportional to $\kappa_{D}^{2}$ [12,13], electrostatic stiffening remains debated [14,15,16,17,18,19]. We will not enter the debate here. Intrinsically stiff biofilaments, which we consider explicitly below, are usually charged and could be termed polyelectrolytes. Under physiological conditions, the electrostatic interaction is strongly screened and short ranged: electrostatic stiffening is negligible. The electrostatic excluded volume associated with the second virial of the screened potential typically does not swell the stiff biofilaments, except for long d-DNA’s. Note however that close to a solution/wall interface, the effective interaction between test-charges is more complicated [20,21,22] than the Yukawa potential given above.

Many recent efforts were directed towards the dynamics of semiflexible chains, e.g., in flows [23,24] or the growth of biofilaments. The former topic is covered by the contribution of Winkler to this issue [25]. Some aspects linked to the coupling of longitudinal and transverse dynamics at short times, like the propagation of tension, were previously addressed by us and others [26,27,28,29]. The growth of biofilaments, essentially actin, was considered by Carlier [30], Mitchison [31] and modeled by Lacoste [32]. We will not discuss any such dynamical issues here.

Though the paper reviews some of the contributions by the theory and simulation group at Institut Charles Sadron (ICS), it proposes many new aspects, approaches or presentations. Section 2 gives results for the WLC in 2D and 3D. Section 3 is devoted to WLC adsorption on a flat surface. Section 4 treats filaments beyond WLC. Section 5 mentions miscellaneous topics.

## 2. The WLC Model: Some Results in 3D and 2D

### 2.1. Single Chain Properties

The statistical physics of a single WLC is a classical topic of textbooks [1,2,3,7]. We hence do not aim at being exhaustive, but give a few results, which are used later. The WLC is described by the bending Hamiltonian $H=\left(B/2\right)\int ds\kappa^{2}\left(s\right)$ where *κ* is the local curvature and the integral runs over the arc length *s*, along the chain. In 2D, a configuration is determined by the set of angles θ(s) between the tangent to the chain and a reference direction. In particular, the local curvature at curvilinear abscissa *s* is simply κ=dθ/ds. Local bending of a small section of length δs by an angle δθ is penalized by a bending energy cost $\delta H=\left(B/2\right){\left(\delta \theta \right)}^{2}/\left(\delta s\right)$ quadratic in δθ. The distribution of the increment δθ is hence Gaussian. Therefore, its Fourier transform is also Gaussian, $B\left(q\right)=e^{-q^{2}\delta sk_{B}T/\left(2B\right)}$, where *q* is conjugated to δθ. The angular deflection along a finite chain is additive, and the global deflection also follows Gaussian statistics $P\left(q\right)=e^{-q^{2}sk_{B}T/\left(2B\right)}$ for a WLC of length *s*. A widely-used geometrical quantity is the tangent/tangent correlation function C(s)=〈cosθ〉 with the reference θ=0 taken at s=0, C(s) is the real part of ${P|}_{q=1}\left(s\right)$. It is convenient to introduce the persistence length $\ell =B/\left(k_{B}T\right)$, which characterizes the exponential decay of C(s):

(1) $$C\left(s\right)=e^{-s/(2\ell )}\;\left(2D\right)\;,\;C\left(s\right)=e^{-s/\ell }\;\left(3D\right)$$

where the 3D expression accounts for the two transverse directions, which contribute to decorrelate the tangent. Other geometrical quantities like the mean-square end-to-end distance $R_{e}^{2}$ or the radius of gyration $R_{g}$ are, quite generally, obtained as multiple integrals of C(s); $R_{e}^{2}\left(S\right)=\int_{0}^{S}ds\int_{0}^{S}ds^{\prime }C(|s-s^{\prime }\left|\right)$ and $2S^{2}R_{g}^{2}\left(S\right)=\int_{0}^{S}ds\int_{0}^{S}ds^{\prime }R_{e}^{2}(|s-s^{\prime }\left|\right)$. For the 2D WLC case, one obtains:

(2) $$\begin{array}{ccc} R_{e}^{2} & = & 4\ell S-8\ell^{2}\left(1-e^{-\frac{S}{2\ell }}\right)\;,\\ R_{g}^{2} & = & \frac{2\ell S}{3}-4\ell^{2}\left[1-4\frac{\ell }{S}\left(1-\frac{1-e^{-\frac{S}{2\ell }}}{S/(2\ell )}\right)\right]. \end{array}$$

The 3D result is obtained by replacing *ℓ* by ℓ/2. Beyond polymer textbooks, more advanced results were reported. The end-to-end distribution of the 2D WLC has been studied theoretically by Lamprecht et al. [33]. In the limit of 2D SAW, Cardy and Saleur calculated the ratio of the end-to-end distance to the radius of gyration [34]. The competition between stiffness and attraction is not addressed here. It is the topic of the contribution by Janke to this issue [35]. In the dilute regime, a variety of filament structures emerge, including stable knots; further, multi-chain aggregates are discussed. The collapse transition was also studied on a 2D lattice [36,37,38].

### 2.2. Melt of Worm-Like Chains

#### 2.2.1. Simulations of WLC Melts and Lattice Artifacts

##### Generalities

**Figure 1:**
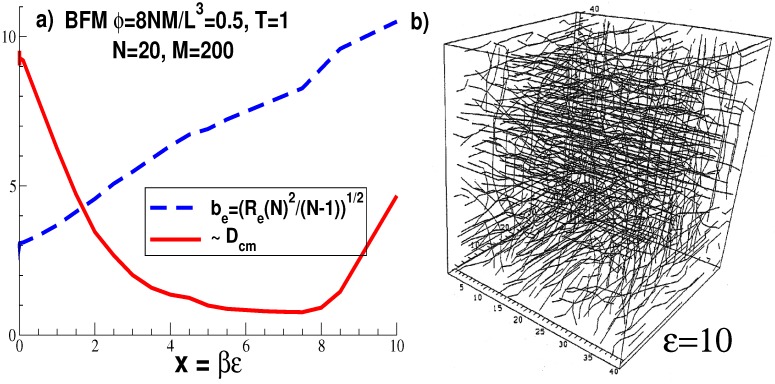
Stiffness effects and lattice artifacts for bond-fluctuation model (BFM) data obtained for M=200 chains of length N=20 at a volume fraction ϕ=8NM/V=0.5 of occupied lattice sites at a temperature T=1/β=1 [41]. Panel (**a**) shows the effective bond length $b_{\text{e}}=R_{\text{e}}\left(N\right)/{\left(N-1\right)}^{1/2}$, obtained from the root-mean-squared chain end-to-end distance $R_{\text{e}}\left(N\right)$ and the (rescaled) center-of-mass self-diffusion coefficient $D_{\mathrm{cm}}$ as a function of the dimensionless parameter x=βϵ. A snapshot of a configuration at ϵ=10 is given in Panel (**b**). The chains are seen to align along the three principal lattice axes.

Semiflexible and rigid polymer chains have also been considered numerically, first [8,39,40,41,42,43,44,45,46,47] using lattice Monte Carlo (MC) simulations [48,49,50,51], where coarse-grained polymer chains are represented by connected lattice sites [43,44]. The monomers move either using local MC jumps between neighboring lattice sites or by means of global collective rearrangements, such as “slithering snake” moves along the chain contour [43]. A particular efficient lattice MC algorithm heavily used in the past is the so-called “bond-fluctuation model” (BFM) [11,52,53,54] where the monomers are represented by cubes on a simple cubic lattice connected by a set of allowed bond vectors. Since a repeat unit on the lattice model represents a group of atoms in a real chain, one can model the chain stiffness by introducing an effective potential that prefers straight conformations. A particular simple choice often used is:

(3) E(α)=ϵcos(α)

with *α* being the complementary angle to the one enclosed by two consecutive bonds [40,41] and *ϵ* the energy parameter allowing one to tune the effective bond length $b_{e}$ as shown by the dashed line in Panel (a) of Figure 1. Most studies start with equilibrated samples of flexible chains (ϵ=0) and increase then gradually the stiffness energy *ϵ*. The equilibration may be checked by comparing with systems obtained starting with rigid rods [50]. 

##### Caveats

We emphasize that the dimensionless parameter x=βϵ (with $\beta =1/(k_{B}T)$ being the inverse temperature) should not become too large, as seen in Figure 1 for a BFM melt of volume fraction ϕ=0.5 with short chains of length N=20 [41]. In fact, the center-of-mass self-diffusion coefficient $D_{\mathrm{cm}}$ levels off at x≈3 and even increases beyond x≈7, albeit the local jump acceptance rate decreases monotonously (not shown). As can be seen from Panel (b) for x=10, chains and chain segments tend to align along the easy lattice axes of the respective model once *x* exceeds unity. It is this breaking of the orientational symmetry of the chains that causes the unphysical non-monotonous behavior of the diffusion constant. The alignment along the easy axes of the model is closely related to the entropically-driven alignment of stiff chains along planar surfaces discussed below. Unfortunately, while the surface alignment is a true physical effect of experimental relevance, the alignment of lattice polymers is merely an algorithmic artifact. We emphasize that this is not an equilibration problem and should also occur if the chains are generated, say, using the pruned-enriched Rosenbluth method (PERM), as in various recent studies [8,47]. Results obtained for very rigid lattice chains [8,45,46,47] may thus be irrelevant for the continuous space experimental reality and should be considered with care. In any case, it is not easy to properly subtract these artifacts, which may be quantified using an orientational order parameter [1] corresponding to a second order Legendre polynomial. In our opinion, future research on semiflexible and rigid chains should thus focus on off-lattice bead-spring models of the Kremer–Grest type [55,56]. While this does a priori not exclude off-lattice MC schemes [51,57,58,59], molecular dynamics has many advantages since collective relaxation pathways are directly excited. Due to the strong correlations of the monomer positions in stiff chains, local jump MC schemes necessarily lead to exponentially long relaxation times (if the chains are not already aligned along an axis) and are thus intrinsically inefficient. Similar anomalous diffusion and chain relaxation are observed for collapsed polymers below the *θ*-point if local MC schemes are used [59].

#### 2.2.2. Two-Dimensional Polymer Melts: Isotropic-Nematic Phase Transition

##### Flexible Chains

As first suggested by de Gennes [60], it is now generally accepted theoretically [61,62,63], numerically [53,64,65,66,67,68] and experimentally [69,70,71], that strictly two-dimensional (D=2) “self-avoiding walks” (SAWs) adopt compact conformations at sufficiently high concentrations *c*, i.e., the chain size:

(4) $$R\overset{!}{\approx }d_{\text{cm}}\approx {\left(N/c\right)}^{\nu }\mathrm{with}\;\nu =1/D=1/2$$

is set by the typical distance $d_{\text{cm}}$ between the chains (the exclamation mark stresses the key scaling assumption). It is assumed here that chain intersections are strictly forbidden. This must be distinguished from systems of so-called “self-avoiding trails” (SATs), which are characterized by a finite chain intersection probability [63]. Relaxing thus the self-avoidance constraint, SATs have been argued to belong to a different universality class revealing mean-field-type statistics with rather strong logarithmic *N*-corrections [63,72]. The SATs’ universality class is relevant to the in-plane chain statistics for thin molten layers [63]. It is important to stress that the compactness of dense, strictly 2D SAWs, Equation (4), does not imply Gaussian chain statistics, since other critical exponents with non-Gaussian values have been shown to matter for various experimentally-relevant properties [61,62,63,66,67]. It is thus incorrect to assume that excluded-volume effects are screened [73] as is approximately the case for 3D melts [11]. Furthermore, the segregation of the chains does by no means impose dislike shapes minimizing the contour perimeter of the (sub)chains; the chains adopt instead rather irregular shapes [53,65,66,67,73,74,75,76]. Focusing on dense 2D melts, it has been shown both theoretically [63] and numerically [66,67,76] that the irregular chain contours are characterized by a perimeter *L* scaling as:

(5) $$L∼R^{D_{p}}∼N^{D_{p}/D}\mathrm{with}\;D_{p}=D-\theta_{2}=5/4.$$

The fractal line dimension $D_{p}$ is set by Duplantier’s contact exponent $\theta_{2}=3/4$ characterizing the size distribution of inner chain segments [62]. We remind that Duplantier’s theoretical predictions obtained using conformal invariance [62] rely on both the topological constraint (no chain intersections) and the space-filling property of the melt. Interestingly, the fractal line dimension is experimentally accessible from the generalized Porod scattering of the intrachain coherent structure factor F(q). It has been shown [66,67] that:

(6) $$NF\left(q\right)\approx N^{2}/{\left(qR\right)}^{2D-D_{p}}\overset{!}{\propto }L$$

should hold in the intermediate wavevector regime.

##### Weak Persistence Length Effects

**Figure 2:**
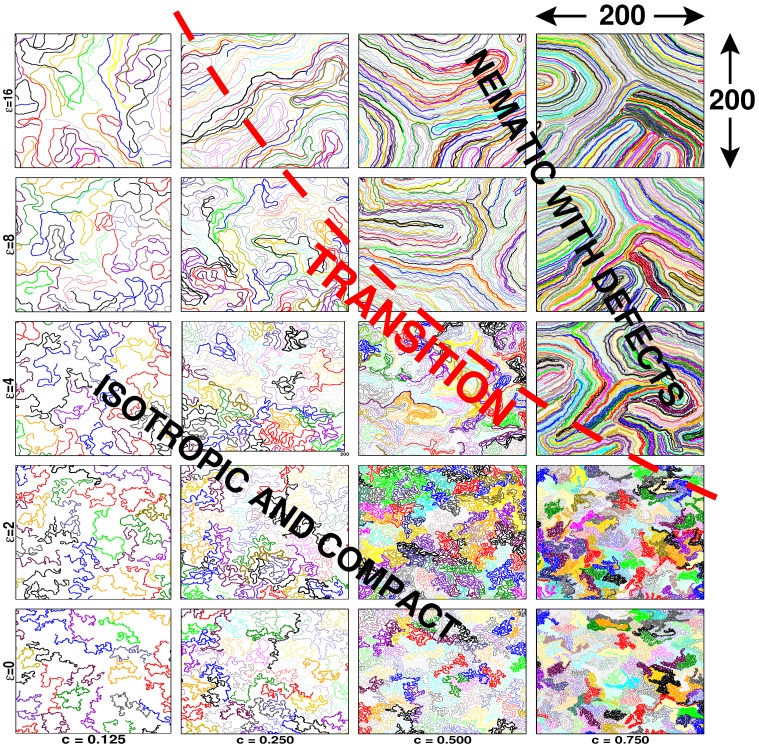
Snapshots of semiflexible 2D polymers of length N=256 obtained by means of molecular dynamics simulation of a Kremer–Grest bead-spring model [56,79]. We show data for four concentrations $c=NM/L^{2}=0.125$ (M=192 chains, linear box size L≈627), c=0.250 (M=192, L≈443), c=0.500 (M=192, L≈313) and c=0.750 (M=384, L≈362) and five bending penalties ϵ=0, 2, 4, 8 and 16 (from the bottom to the top). Only small subvolumes of much larger boxes are represented. The configurations have been sampled by increasing *ϵ* starting with flexible and compact chain systems (ϵ=0) [68,76,80]. While the chains remain compact and segregated at low densities and stiffnesses below the dashed line, they are seen in the opposite limit to align (at least) locally, forming bundles of chains with hairpins, which are extremely difficult to equilibrate.

**Figure 3:**
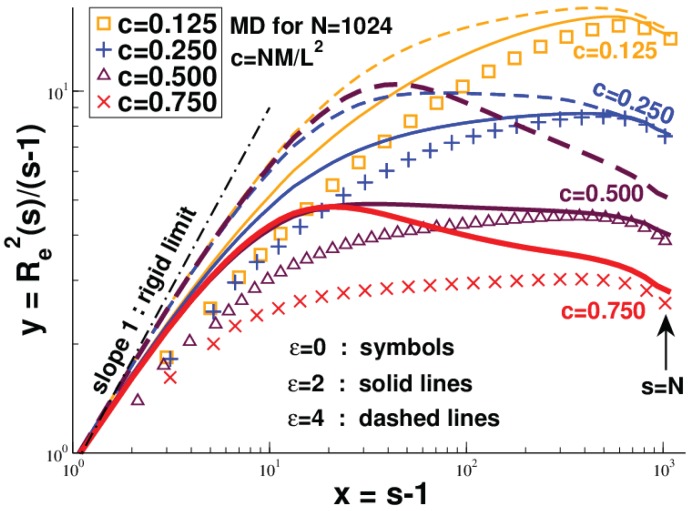
Rescaled sub-chain size $y=R_{\text{e}}^{2}\left(s\right)/\left(s-1\right)$ with *s* being the arc-length (sub-chain length) for four concentrations and three stiffness penalties corresponding to systems below and around the dashed line in Figure 2. The symbols refer to flexible systems (ϵ=0); the line width for ϵ=2 and 4 increases with density. The dash-dotted line corresponds to the asymptotic slope for perfectly rigid chains. Note that y(s) becomes strongly non-monotonous with increasing *ϵ*. However, for a given density and s→N, all y(s) become similar as long as the system remains isotropic, i.e., *ϵ* is not too large. Independent of the rigidity, the overall chain size is thus ruled by the (persistence length independent) distance $d_{\text{cm}}\approx {\left(N/c\right)}^{1/D}$ between chains.

Up to now, we have supposed that the chains are flexible down to the monomer scale. Obviously, most of the experimentally-relevant macromolecules on surfaces or confined to thin films are rather rigid [69,70,71,77,78]. Following previous computational work [8,39], we are currently re-investigating the scaling of semiflexible chains starting from the dilute limit [8] up to high densities to see how a finite persistence length may change the scaling. Semiflexibility is readily included by adding the coarse-grained stiffness potential Equation (3) to the widely-used Kremer–Grest bead-spring model [56,79]. The effect of this potential is best first characterized for the dilute limit, which yields the curvilinear persistence length from the exponential decay of the bond-bond correlation function following Hsu et al. [8]. Note that the bond length *l* can be regarded as constant for all *ϵ* and *c*. It is found that the persistence length remains smaller than the semidilute blob size ξ(ϵ) for the experimentally mainly relevant low and moderate densities. In this regime, the finite rigidity is observed not to change the scaling properties for the flexible chains discussed above. This may be seen simply from the snapshots below the dashed line presented in Figure 2. In fact, since the number of monomers *g* in the semidilute blob decreases rapidly with *ϵ* [68], i.e., N/g increases strongly as long as *g* does not become too small (g≫1), a finite rigidity even speeds up the convergence to the predicted compact chain limit. As shown from the reduced sub-chain size $R_{\text{e}}^{2}\left(s\right)/\left(s-1\right)$ for different *ϵ* presented in Figure 3, the sub-chain size depends in fact very little on the local persistence length for large arc-lengths s→N. Essentially, the chain size is set in this limit by the *ϵ*-independent distance $d_{\text{cm}}$, cf. Equation (4), between chains.

##### Strong Persistence Length Effects

If the persistence length exceeds strongly the blob size (estimated by extrapolation to finite densities using the conformational properties of the dilute limit), with increasing persistence length and density, one expects to observe a transition to an ordered nematic state. The location of this transition is roughly sketched by the dashed line in Figure 2. Whether this transition is of continuous order, as claimed in some theoretical studies focusing on semiflexible polymer chains of a fully-occupied Flory lattice model confined to a volume of fixed shape [45,46], or of first order, as suggested by the Monte Carlo simulations of multi-chain systems at a fixed finite density [39], is an important and, in our view, yet open question. The data presented in Figure 2 and Figure 3 stem from systems where we start with compact conformations of flexible chains (ϵ=0) and increase gradually the bending penalty. Additionally, a second set of data has been obtained starting with aligned rods at high *ϵ* decreasing then gradually the stiffness (this dataset is limited up to now to short chains of length N≤256). While for systems below the dashed line in Figure 2, the same chain conformations are readily reached with both protocols for all chain lengths available, this becomes computationally challenging around and above the dashed line, i.e., strong hysteresis effects have been observed. Due to the sluggish hairpin-like defects seen in the snapshots, the hysteresis might be a merely dynamical issue. However, for all systems for which both protocols yield the same configurational properties, i.e., where we are sure to have reached equilibrium, a sharp transition between isotropic and nematic states has been observed for the standard nematic order parameter *S* [1], the reduced chain size $R_{e}^{2}\left(N\right)/N$ or the second Legendre polynomial $P_{2}\left(r\right)$ of intra- and inter-chain bonds as a function of distance *r* between bonds (not shown). These results point to a first order transition [51], supporting thus the pioneering work by Baumgärtner and Yoon [39]. From a mathematical point of view, the associated 2D lattice models [45,46] are solved exactly. The same system has also been studied on a different lattice more recently [81]; there, a Kosterlitz–Thouless (KT) transition is reported. Experimentally and in simulations, the KT transition is most often hidden by a first order transition induced by short range interactions.

#### 2.2.3. Three-Dimensional Polymer Melts: Corrections to Chain Ideality

As pointed out in the Introduction, away from the isotropic/nematic transition, rigidity effects are less important in three-dimensional (3D) concentrated solutions or polymer melts, and the classical view about flexible polymer chains should apply. This classical view supposes that all spatial correlations are short-range: they vanish for length scales larger than the correlation length characterizing the decay of the density fluctuations [1,2,7,9]. However, over the past years, there has been increasing evidence that this classical view must be amended. For instance, the correlation functions of density fluctuations [82] or bond orientations [10,11] display long-range power law decays. At the single chain level, this implies corrections to ideal (Gaussian) chain statistics. The molecular origin of these deviations from chain ideality is related to the interplay of chain connectivity and the incompressibility of the melt. This interplay leads to an effective repulsion between chain segments due to a correlation hole effect, leading to $C\left(s\right)∼s^{-3/2}$ for the tangent/tangent correlation function for large arc-length *s* [10,11].

**Figure 4:**
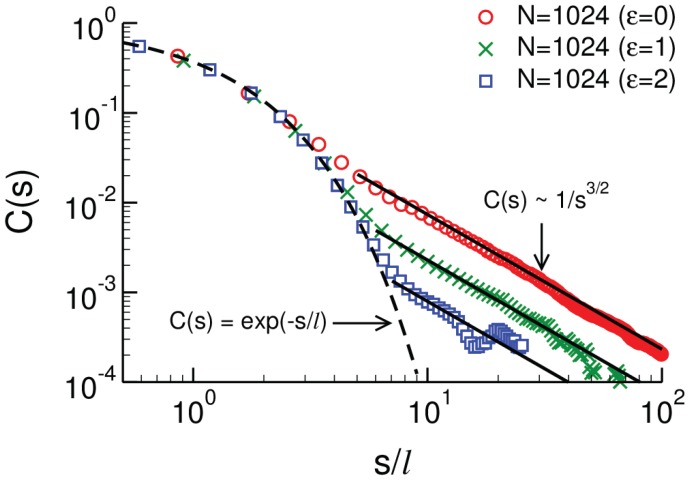
Log-log plot of the tangent/tangent correlation function C(s)=〈cosθ(s)〉 versus arc-length *s* for a 3D polymer melt with chains of length N=1024. The symbols show data from MD simulations for a Kremer–Grest-like bead-spring model [10] with three bending penalties: ϵ=0,1,2. The model is similar to the one shown in Figure 2. Thus, the melt with ϵ≤2 is isotropic, the value ϵ=0 corresponding to fully-flexible chains. The abscissa is scaled by the persistence length *ℓ* obtained from a fit of the initial decay of C(s) to Equation (1); see the dashed line in the figure. The solid lines indicate the power law, $C\left(s\right)∼s^{-3/2}$, expected from corrections to chain ideality [10,11].

Since the corrections should manifest themselves best for (nearly) incompressible systems of long flexible chains, our numerical tests by simulations have employed fully-flexible generic polymer models, e.g., the bond-fluctuation lattice model (BFM) and a Kremer–Grest-like bead-spring model (BSM) [10]. As an example, Figure 4 depicts C(s) for the BSM and chains of length N=1024. The power-law $C\left(s\right)∼s^{-3/2}$ is clearly visible for the fully-flexible model (ϵ=0) and also persists, if rigidity is switched on (see the data for ϵ=1 and 2), although the amplitude of the power law is decreased. A weak rigidity was recently suggested to be a good compromise if one wants (to simulate) pretty flexible chains with weak non-ideality effects [83]. 

## 3. Adsorption of WLC

When a polymer can bind to a surface by any of its monomers, it tends to do so against translational and conformational entropy, a process called adsorption. Adsorption from solution is a very efficient coating process and an amount equivalent to a few monolayers of monomers is usually enough to control surface properties. Several numerical studies describe polymer adsorption with rigidity [84,85]. The adsorption process turns out to be rather complex. We focus on two extreme cases, reversible adsorption where the layer is in full equilibrium with the bulk solution [86,87] and chemisorption where polymer binds through irreversible monomer/wall chemical reaction [88,89]. In the former case, adsorbed polymer can reorganize freely and exchange freely with the bulk, whereas in the latter case, an established monomer/wall bond is permanent, and the polymers can only reorganize under this strong constraint and never desorb. Though these two adsorption processes are at the extreme of a spectrum extending from fully-equilibrated to totally-irreversible processes, it turns out that their gross features can be described with the same tools.

### 3.1. Loop and Tail Partition Function

Adsorption of a chain produces configurations that can be analyzed in terms of loops and tails [90]. Loops are chain fragments that touch the surface only by their ends, the configuration comprises a series of loops. Sometimes, in discrete models (say, if space is described by a discrete lattice), a series of monomers laying “flat” on the surface is called a train [90]. A tail is an end-section leaving the surface without touching it again; there are two tails per adsorbed chain.

The equilibrium distribution of loops and tails plays an essential role and turns out to rule over the structure of the adsorption layer for both reversible adsorption and chemisorption. Actually, the polymer statistics merely enters the adsorption problem via these distributions. In particular, the loop and tail distributions encode for the quality of the solvent and, more importantly here, the chain stiffness. In line with Section 1, we neglect excluded volume interactions between persistent segments and restrict our attention to ideal loops and tails. We anticipate that fragments (loops or tails) shorter than *ℓ* are stiff, while larger ones are flexible and follow Gaussian statistics.

Let us first notice that bringing a chain in contact with a repulsive flat surface by any of its monomers does not affect its configurations. To see this in a simple way, take an arbitrary bulk configuration and translate it down to the wall until the closest monomer comes into contact. Any bulk configuration generates one configuration touching the wall by any of its monomers, the reverse being obviously true. The equivalence of the two sets of configurations translates into simple relations on partition functions, which were derived in many specific cases, e.g., for excluded volume chains, using dedicated methods [62].

Take a flexible chain in the bulk; omitting the exponential contribution associated with monomer fugacity, its partition function can be set to unity. The same holds true for a chain touching the wall by any of its *N* monomers. Take now a chain specifically touching with one of the ∼N middle monomers, its partition function is $Z_{1}∼1/N$. The touching configuration can be seen as made out of two tails; hence, the tail partition function satisfies $Z_{t}^{2}∼1/N$ and $Z_{t}\left(n\right)∼1/\sqrt{n}$; a standard result that is easily recovered from other methods, for example, from the image principle. A loop of size *n* can be considered as made out of two tails of size ∼n, which are within reach and with ends constraint to match in height [91]; hence, the loop partition function satisfies $Z_{l}\left(n\right)∼Z_{t}{\left(n\right)}^{2}/\sqrt{n}$ and $Z_{l}\left(n\right)∼n^{-3/2}$, as anticipated from first passage statistics. The advantage of this derivation over more standard ones is that it easily generalizes to semiflexible chains. Below, we use a very similar way to derive partition functions of stiff loops and tails [92,93].

**Figure 5:**
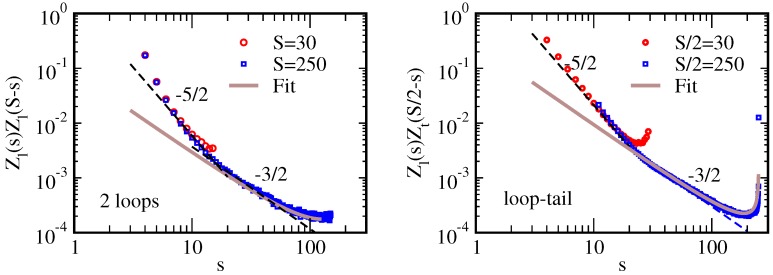
Loop distribution as simulated by molecular dynamics for two chain lengths (S=30 b and S=250 b). The persistence length is ℓ=10.1 b throughout. (left) Distribution of the internal loop size upon first adsorption of a loop of size *S*; only the smallest of the generated internal loops is taken into account; the full distribution is symmetric about S/2. We show the power law fit by the single loop partition function (dashed lines) for the stiff loop and for the flexible loop, which apply where they should. Note the rather narrow crossover around s=2ℓ. The product of loop flexible partition functions nicely accounts for the flattening near s=S/2 required by symmetry. (right) Distribution of the size of the loop generated by first re-adsorption of a tail of length S/2. Again, the single loop partition functions fit where they should; the narrow crossover is now located around s=ℓ. The product of the flexible loop and tail partition functions accounts for the upturn near s=125 b where the small tail partition function dominates.

Noticing that for a stiff bulk chain of size *s*, the typical global angular fluctuation *θ* is given by $\ell \theta^{2}/s∼1$ leading to $\theta ∼\sqrt{s/\ell }$, the chain partition function can be equated to this fluctuation. The partition function of a stiff chain touching the surface by any of its monomers is hence also $Z_{1}\propto \sqrt{s}$. The stiff chain touching the surface by a specific middle monomer can now be seen as composed of two tails of length ∼s, $Z_{1}/s\propto Z_{t}^{2}$, solved by $Z_{t}\propto s^{-1/4}$. We can then derive the partition function of a stiff loop of size *s*: a stiff loop is composed of two stiff tails matching both in orientation and height. The orientation and height of each tail are fluctuating within $\theta ∼\sqrt{s/\ell }$ and z∼sθ, respectively. The partition function of the loop as a constraint state of the two tails reads $Z_{l}∼Z_{t}^{2}/\left(\theta z\right)$. This leads to $Z_{l}\propto s^{-5/2}$. Although this derivation is similar to the one for a flexible chain, the additional constraint on the orientation had to be implemented. Loop distributions obtained from numerical simulation are shown in Figure 5 together with the predicted asymptotic laws [93].

### 3.2. Reversible Adsorption of an Ideal WLC from Dilute Solution

We may proceed with a simple description of the adsorption of ideal WLC from solution and discuss some of the many additional subtleties in a second step. The very vicinity of the surface z<ℓ, the so-called proximal layer, is populated with short stiff loops. At distances from the wall smaller than *ℓ*, stiff loops build up the concentration c(z). Their size *s* is distributed according to the partition function $Z_{l}\propto s^{-5/2}$. These stiff loops are pretty flat and extend into the solution over a distance $z∼s^{3/2}/\ell^{1/2}$ according to their size. The sublayer $z<\hat{z}$ is merely filled with loops of size $s<\hat{s}∼{\hat{z}}^{2/3}\ell^{1/3}$, which translates into:

(7) $$\int_{0}^{\hat{z}}c(z)dz=\int_{0}^{\hat{s}}Z_{l}\left(s\right)sds$$

Taking the derivative with respect to $\hat{z}$ on both sides of Equation (7), we obtain the concentration profile:

(8) $$c\left(z\right)\propto z^{-4/3}$$

Although this result reminds the celebrated self-similar concentration profile obtained by de Gennes for the adsorption of swollen chains from a good solvent [94], the latter decreases with an exponent, which is only approximately 4/3 in 3D and depends on the dimension of space, it is unrelated. For strong enough adsorption, the adsorption layer is thin and limited to the proximal regime Equation (8) with a proper cut-off function defining its thickness *h*.

For ideal chains the critical adsorption strength depends on the range Δ of the adsorption potential $u_{c}∼1/{\left(\Delta^{2}\ell \right)}^{1/3}$ [92,95] that reflects the balance between Odijk confinement energy [14] and the gain in interaction energy. Strong adsorption with a layer thickness *h* shorter than *ℓ* is found far enough from the adsorption threshold, for reduced temperatures $\tau =\left(u-u_{c}\right)/u_{c}$ larger than ${\left(\Delta /\ell \right)}^{2/3}$, a regime where $h∼\Delta /\tau^{3/2}<\ell$. In practice, the adsorption layer is typically thin and described by Equation (8) up to the cut-off. A very detailed analytical description of the proximal layer build by ideal WLC is proposed in [92] together with an analysis of weak adsorption. In the weak adsorption regime, the concentration profiles are found to be non-monotonic and to go through a local minimum. The work in [92] proceeds from an eigenstate description of the partition function [1]. The boundary condition is rather subtle and basically ensures that no fragment is coming out of/penetrating into the wall, it is neither a von Neumann, nor a Dirichlet boundary condition. Earlier attempts [96,97,98] failed due to improper boundary conditions, which led to a ground state with a node, which cannot be.

What is the impact of excluded volume on the layer description? As long as the chain thickness *b* is small compared to the persistence length, b≪ℓ, the power law decay of the concentration in the proximal regime Equation (8) must hold. Excluded volume can become important in the concentrated quasi-2D layer within the adsorption well and may affect the adsorption transition. A recent single mushroom adsorption study by numerical simulation [47] finds that the critical adsorption energy varies with the persistence length as $u_{c}\propto 1/\ell$. It is fair to say that the numerical study of critical adsorption is very difficult and was performed on a cubic lattice in [47], which affects the powers of Δ/ℓ in various scaling behaviors, e.g., for $u_{c}$ (see also the remark in the final discussion in [92]). Recent numerical self-consistent mean-field calculations in Chen’s group [99] are compatible with the standard predictions for $u_{c}$ [92,95], as they should be. Excluded volume can also affect the flexible loop layer in the weak adsorption regime, but merely for very large loops (see Section 1).

### 3.3. Irreversible Chemisorption of a WLC from Dilute Solution

Chemisorption is a very slow adsorption process limited by the kinetics of the chemical reaction, which ensures binding. Polymer configurations are equilibrated at every step under the constraint of already existing bonds. In this sense, the adsorption can be looked at as an Eyring reaction. Under this condition, the reaction rate defined as the number of surface bonds created per unit time and area *Q* decomposes into the product of the (intrinsic) reaction rate (frequency) *q* for monomers within the reaction distance from the surface and the concentration of monomers $c_{0}$ within the reaction distance (z<b). The latter is to be calculated for polymer configurations equilibrated under the constraints imposed by already bound monomers [100,101,102,103]. Below, we consider chemisorption from a dilute solution. Among the relevant questions are: given that a polymer has established a first bond with the surface, how does adsorption proceed further? Will a single chain zip on the surface out of the first bond or will zipping be preempted by the formation of large bound loops accelerating the adsorption process? What layer structure will the chemisorption process ultimately build? The latter is of prime practical importance. Adsorption kinetics can also be addressed.

Suppose one middle monomer is bound flat on the surface; because of chain stiffness, neighboring monomers stay merely within the reaction distance ∼b. The section that is flat within the distance *b* has the typical length $s_{0}∼{\left(\ell b^{2}\right)}^{1/3}$, longer sections do thermally fluctuate out beyond z∼b. A monomer, which is a chemical distance $s<s_{0}$ away from the first bond, is hence also within the reaction distance with a probability P(s)∼1. For more distant monomers where $s>s_{0}$, $P\left(s\right)\propto Z_{l}\left(s\right)Z_{t}\left(S/2-s\right)/Z_{t}\left(S/2\right)$. Provided s≪S/2, only the loop partition function matters. Assuming a stiff loop s≪ℓ for which $Z_{l}∼s^{-5/2}$, the probability P(s) is proportional to $s^{-5/2}$. To match P(s)∼1 for $s=s_{0}$, we must have $P\left(s\right)∼{\left(s/s_{0}\right)}^{-5/2}$ for $\ell >s>s_{0}$. Even more distant monomers where S/2≫s>ℓ contact the wall by a flexible loop for which $Z_{l}∼s^{-3/2}$ and $P\left(s\right)\propto s^{-3/2}$, matching this and the preceding regime at s=ℓ imposes $P\left(s\right)∼{\left(\ell /s_{0}\right)}^{-5/2}{\left(s/\ell \right)}^{-3/2}$. The three regimes for the monomer wall “contact” probability are summarized below:

(9) $$\begin{array}{cc} P(s)∼1 & (s<s_{0});\\ P(s)∼{\left(\frac{s}{s_{0}}\right)}^{-5/2} & (\ell >s>s_{0});\\ P(s)∼{\left(\frac{\ell }{s_{0}}\right)}^{-5/2}{\left(\frac{s}{\ell }\right)}^{-3/2} & (S\gg s>\ell ); \end{array}$$

with $s_{0}∼{\left(\ell b^{2}\right)}^{1/3}$. These relations were derived for the adsorption of a tail, the same relations do hold for the internal adsorption inside a loop. The cumulated adsorption rate of the single tail (or loop) $Q_{s}∼\int_{0}^{S}qP\left(s\right)ds$ is dominated by $s∼s_{0}$. This indicates that the typical adsorption loop is a small flat loop of size $∼s_{0}$, which we call the minimal loop. Adsorption out of the existing bond hence proceeds by zipping with finite steps of size $s_{0}$ and a rate in steps per unit time $qs_{0}/b$. This is an effect of stiffness, would the chain be flexible, zipping would proceed monomer by monomer. Next question is whether zipping can be completed before a large adsorption loop forms. The zipping process is completed to the length *s* after the time $t_{z}∼{\left(s_{0}q/b\right)}^{-1}\left(s/s_{0}\right)$. The rate of nucleation of an adsorption loop larger than *s* is given by $Q_{n}∼\int_{s}^{S}\;qP(s)ds/b$. Nucleation of a stiff loop (ℓ>s) occurs with a rate $Q_{n}∼q\left(s_{0}/b\right){\left(s/s_{0}\right)}^{-3/2}$, while the nucleation rate for a large flexible loop (S>s>ℓ) is $Q_{n}∼q\left(\ell /b\right){\left(\ell /s_{0}\right)}^{-5/2}{\left(s/\ell \right)}^{-1/2}$. Note that the nucleation of a loop larger than *s* is dominated by the nucleation of loops of size ∼s. Calculating the probability to nucleate a loop larger than *s* before the zipping time associated with length *s*, $t_{z}\times Q_{n}$ shows that large loop nucleation is negligible in the stiff regime s<ℓ (provided $s>s_{0}$) and only preempts zipping deeper inside the flexible regime for $s>S^{*}$ with $S^{*}∼\ell^{5/3}/b^{2/3}$. Table 2 gives the order of magnitudes for the length scales involved in single WLC adsorption.

At this stage, the length $s_{0}$ appears to play a special role, WLC much smaller than $s_{0}$ are hyper rigid; they hardly bend over each other in the multi-chain problem. One chain adsorbs at once (or not at all). We feel that this regime, which we did not study, shares some features with the random sequential adsorption (RSA) of perfectly stiff rods, which only adsorb without overlap. Ultimately, the very stiff WLC should cross over to the RSA regime studied by Viot and Tarjus in [104,105]; it leads to a fractal 2D coverage. This regime may well be pertinent to fragments of the stiffest biofilaments in practice.

What happens if chains adsorb from a dilute solution? Suppose one pretty stiff chain is already adsorbed and another one is coming in from the solution, attaches by one middle monomer, which is most likely, and starts to zip. Let us assume that the configurations are almost straight lines, and if the chains were ideal, they would just cross. The orientations of the two lines are chosen randomly, yet given they cross, their relative orientation is not random; parallel lines do not cross. It is easy to see that the distribution of the crossing angle *θ* is given by P(θ)=(sinθ)/2 with 0<θ<π. The most likely cutting angle is π/2, and the distribution is symmetric about it, while small angles being suppressed from the distribution. The probability to observe an angle smaller than *θ*, is $P_{<\theta }=\theta^{2}/4$ for θ≪1, which we may double by symmetry.

Now, consider a non-ideal filament. It will freely zip until zipping is hampered by the already adsorbed line, which is at a distance along the filament of order $∼s_{0}$. According to the partition function, the filament may now either cross by a loop of size $∼s_{0}$ or bend over and align with the already adsorbed line. Bending over by an angle *θ* is associated with an energy penalty $∼\ell \theta^{2}/s_{0}$, which is irrelevant if *θ* happens to be smaller than $\theta_{\max}<{\left(b/\ell \right)}^{1/3}$, which occurs with the probability $P_{<\theta_{\max}}∼{\left(b/\ell \right)}^{2/3}$. Typically, a given crossing should be preferred to alignment. Now, a given chain experiences a number of crossings, especially the later adsorbing ones. The question whether the chain prefers to cross or align is meaningful when the typical spacing between crossings, which are Poisson distributed, is at least larger than $∼s_{0}$. A typical chain experiences less than $S/s_{0}$ crossings and is unlikely to align during adsorption provided it is stiff $s_{0}<S<\ell$. This simple reasoning leaves open a number of questions. If it so happens that a chain aligns, in how much does this favor alignment of the next coming chain or how does an alignment defect grow when new chains enter the surface? Even if alignment would not be typical, alignment defects must appear in a macroscopic sample. In practice, part of these issues may actually depend on the local mechanism of flexibility. The adsorption loop size is power law distributed; there are scarcer adsorption loops larger than $s_{0}$, which can bend over more easily. The crossover between crossing and aligning around $\theta_{c.o.}∼\theta_{\max}$ is smooth. For moderate rigidity, $\theta_{\max}$ is not that small, and alignment is not strongly suppressed. These expectations are in part born out in preliminary numerical simulations [106] studying the crossing vs. alignment statistics as a function of the orientation *θ* of the incoming filament with respect to the adsorbed one. Simulations also suggest that the problem might be more complex than suggested by the simplistic arguments given above.

In [93], we address irreversible adsorption without alignment in some detail. Overall stiff chains first build a Poisson array of almost straight lines characterized by its length concentration per unit area $c_{2}b$ fixed by the surface concentration $c_{2}$. The distribution of the distance between crossings along a particular line *ξ* decays exponentially with an average *ξ* given by the inverse length density per unit area $\xi ∼1/c_{2}b$. When *ξ* goes below $s_{0}$, adsorption in the typical holes is penalized by an energy barrier against bending, and available holes become scarce. A layer of larger yet stiff loops form, jumping straight from one residual hole to another. These loops build a concentration profile c(z)∝1/z. At a later stage, somewhat larger loops, which explore a somewhat wider lateral region, build a concentration profile $c\left(z\right)\propto 1/z^{2}$. Finally, more separated holes are spanned by flexible loops again building a profile c(z)∝1/z. To summarize:

(10) $$\begin{array}{cc} c(z)∼\frac{c_{2,\infty }}{z} & (z<\frac{1}{c_{2,\infty }b});\\ c(z)∼\frac{1}{bz^{2}} & (\ell >z>\frac{1}{c_{2,\infty }b});\\ c(z)∼\frac{1}{b\ell z} & (\sqrt{S\ell }>z>\ell ); \end{array}$$

with $c_{2\infty }$ the ultimate 2D monomer concentration. Ultimately, remaining holes cannot be spanned by a chain and are occupied by single grafted chains building the outermost layer.

## 4. Filaments in 2D beyond WLC

A number of experimental observations on biofilaments cannot simply be accounted for by the WLC model. For example, the circularization of short actin filaments is observed [107], which would be absent for a WLC with the measured bending rigidity ℓ≈17μm of actin. Similarly, taxol-stabilized microtubules display “intrinsic curvature” unexpected for a stiff WLC. They display length-dependent persistence length [108] when naively considered as WLCs, and their transverse relaxation time scales as the filament length cubed [109]. Finally, microtubules curl-up both in vivo [110,111] and in vitro [112,113] under moderate forces.

These and more puzzles call for augmented models beyond the WLC. Rather than going into the details of the specific biological situations, which is beyond the scope here, we will review some generic model ideas (namely helicity, bistability and polymorphic transitions) and discuss the resulting behavior from the theoretical point of view.

### 4.1. The Helical Filament Squeezed in 2D

**Figure 6:**
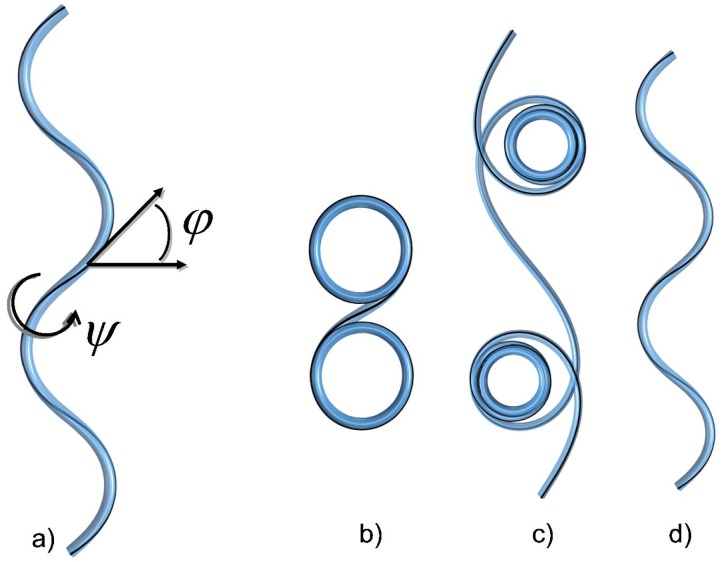
(**a**) Schematic squeelix with the angle *ϕ* that is slaved to the twist angle *ψ* given by the line in black; (**b**) typical shape of a squeelix for γ=1 (see Equation (19)) with a single twist-kink; (**c**) a squeelix in the dilute regime of twist-kinks with γ=0.997; (**d**) a squeelix in the dense regime of twist-kinks with γ=0.52. The ground states (b), (c) and (d) are from [121].

Helically-coiled filaments are a frequent motif in nature. Plenty of biological filaments (FTsZ [114], Mrb [115], basterial flagella [116], tropomyosin [117], intermediate filament vimentin [118]), as well as artificial man-made materials (e.g., micelles [119]) have a helical shape in 3D space. Even whole microorganisms exhibit helicity inherited from their constituent filaments [120]. In situations commonly encountered in experiments, coiled helices are observed when they are squeezed flat onto a two-dimensional surface that coincides with the focal plane. This confinement changes the physical properties of the underlying objects and peculiar squeezed conformations often resembling looped waves, spirals or circles, cf. Figure 6, are observed. Here, we will rationalize the various shapes of those squeezed helical filaments (coined squeelices in the following) and also consider their statistical mechanics properties.

#### 4.1.1. A Bit of Mechanics

Helical worm-like chain model: To describe the shape of an elastic rod of length L, one can use (cf., e.g., [122]) the Frenet–Serret basis (n,b,ø) attached to the rod’s centerline parametrized by the arc-length *s*. In general, such a rod can have an internal twist, which can be taken into account by considering another local basis $\left(e_{1},e_{2},e_{3}\right)$, which rotates with the material. This material frame or director basis $\left(e_{1},e_{2},e_{3}\right)$ is such that $e_{3}=ø$, $e_{1}=n\cos\psi +b\sin\psi$ and $e_{2}=-n\sin\psi +b\cos\psi$ where *ψ* is the twist angle. For a given spatial configuration of the filament, the evolution of this basis along the arc-length centerline is given by the twist equations $e_{i}^{\prime }=\Omega \left(s\right)\times e_{i}$, where $\Omega \left(s\right)=\left(\Omega_{1},\Omega_{2},\Omega_{3}\right)$ is the strain vector and ${\left(\right)}^{\prime }$ denotes the derivative with respect to s. The components of **Ω** are [123]:

$$\begin{array}{cc} \Omega_{1}\left(s\right) & =\kappa (s)\sin\psi (s)\\ \Omega_{2}\left(s\right) & =\kappa (s)\cos\psi (s)\\ \Omega_{3}\left(s\right) & =\tau \left(s\right)+\psi^{\prime }\left(s\right) \end{array}$$

The local curvature is therefore $\kappa^{2}\left(s\right)=\Omega_{1}^{2}+\Omega_{2}^{2}$, and the twist density $\Omega_{3}\left(s\right)$ is the sum of a torsion and the excess twist $\psi^{\prime }.$ The energy $E[\left\{\Omega (s)\right\}]$ of a filament is a functional of the strain vector Ω(s) that can be expanded à la Ginzburg–Landau:

$$E=\sum_{ij}A_{ij}\int \Omega_{i}\Omega_{j}ds+\sum_{ijk}D_{ijk}\int \Omega_{i}\Omega_{j}\Omega_{k}ds+...$$

The shape of the filament at zero temperature will be obtained my minimizing the elastic energy δE/δΩ(s)=0.In the linear elastic regime, where the stress **σ** is proportional to the strain, i.e., σ(s)=δE/δΩ(s)∝ Ω(s), the expansion stops at the quadratic order in **Ω**. We limit ourselves to this regime for the moment. See the Section 4.2.2 on filament crunching for nonlinear contributions.

The linear elasticity of an isotropic rod: The straight untwisted rod minimizes the energy:

$$E=\frac{1}{2}\int ds\left(B\kappa^{2}\left(s\right)+C\Omega_{3}^{2}\right)$$

with *B* and *C* the bending and torsional stiffness, respectively. Now, a helical elastic rod of a circular cross-section is a curve with constant curvature and torsion. Therefore, its elastic energy is of the form:

(11) $$E=\int \left[\frac{B}{2}\left({\left(\Omega_{1}-\omega_{1}\right)}^{2}+{\left(\Omega_{2}-\omega_{2}\right)}^{2}\right)+\frac{C}{2}{\left(\Omega_{3}-\omega_{3}\right)}^{2}\right]ds,$$

with $\omega_{1}$ and $\omega_{2}$ the principal intrinsic curvatures and $\omega_{3}$ the intrinsic torsion. These material constants are chosen as positive. Upon a proper definition of the material frame, one can always set $\omega_{2}=0$. In the 3D situation, one can minimize the bending and the twist parts of the energy given by Equation (11) independently, yielding a curve of constant curvature $\omega_{1}$ and torsion $\tau =\omega_{3}.$ In the absence of external torque, there is no excess twist, $\psi^{\prime }=0$. This 3D ground state corresponds to a helix of radius $R=\frac{\omega_{1}}{\omega_{1}^{2}+\omega_{3}^{2}}$ and pitch $H=\frac{2\pi \omega_{3}}{\omega_{1}^{2}+\omega_{3}^{2}}$ satisfying the preferred curvature and twist everywhere. To proceed further, it is convenient to introduce the Euler angles $\phi \left(s\right),$ $\theta \left(s\right)$ and $\psi \left(s\right)$, such that the components of the strain vector are given by:

$$\begin{array}{cc} \Omega_{1} & =\phi^{\prime }\sin\theta \sin\psi +\theta^{\prime }\cos\psi \\ \Omega_{2} & =\phi^{\prime }\sin\theta \cos\psi -\theta^{\prime }\sin\psi \\ \Omega_{3} & =\phi^{\prime }\cos\theta +\psi^{\prime } \end{array}$$

In this formulation, the curvature is given by $\kappa^{2}\left(s\right)=\phi^{\prime 2}\sin^{2}\theta +\theta^{\prime 2}$, and the torsion is $\tau =\phi^{\prime }\cos\theta .\;$

Ground state of a squeelix: Confining the helical rod on the plane amounts to put θ=π/2 and then:

$$\Omega_{1}=\phi^{\prime }\sin\psi ,\;\;\;\Omega_{2}=\phi^{\prime }\cos\psi ,\;\;\;\Omega_{3}=\psi^{\prime }.$$

Hence, the local curvature is simply given by $\kappa^{2}\left(s\right)=\phi^{\prime 2}$, and torsion τ=0, as the curve is now planar. The energy of a squeelix of length *L* then reads:

(12) $$E=\frac{1}{2}\int_{-L/2}^{L/2}B\left(\phi^{\prime 2}-2\omega_{1}\phi^{\prime }\sin\psi +\omega_{1}^{2}\right)+C{\left(\psi^{\prime }-\omega_{3}\right)}^{2}ds$$

Note that under confinement, the curvature and twist are now coupled. The energy variation δE=0 leads to the following Euler–Lagrange equations:

(13) $$\phi^{\prime }=\omega_{1}\sin\psi$$

(14) $$\psi^{\prime \prime }+\frac{B\omega_{1}^{2}}{2C}\sin\left(2\psi \right)=0$$

and the boundary conditions:

(15) $$\psi^{\prime }\left(-L/2\right)=\omega_{3}=\psi^{\prime }\left(L/2\right)$$

Thus, even in the absence of an external torque at the chain’s end, the confinement converts the intrinsic torsion into an intrinsic torque. Equation (14) is nothing but the pendulum equation (with arc-length *s* replacing time) whose solution depends on the material parameters B,C, $\omega_{1}$ and also $\omega_{3}$ through the boundary condition Equation (15). The solution of this equation leads to the curvature $\kappa \left(s\right)=\phi^{\prime }\left(s\right)$, which is slaved to the twist according to Equation (13). This is the most important consequence of the squeezing of a helical WLC.

From the knowledge of $\phi^{\prime }\left(s\right)$, the 2D shapes can be reconstructed from the Cartesian coordinates given by:

(16) $$\begin{array}{cc} x(s) & =x_{0}+\int_{-L/2}^{s}\cos\left(\phi (s^{\prime })\right)ds^{\prime } \end{array}$$

(17) $$\begin{array}{cc} y(s) & =y_{0}+\int_{-L/2}^{s}\sin\left(\phi (s^{\prime })\right)ds^{\prime }. \end{array}$$

The solutions that minimize the elastic energy of the squeelix lead to a variety of shapes resembling loops, waves, spirals or circles (see Figure 6).

Solving Equation (14) for a chain of finite length *L* with Equation (15) turns out to be surprisingly complicated [121]. To grasp a physical intuition of the squeelix from simple arguments, we consider a very long chain with *L* much larger than any characteristic length and neglect the boundary condition Equation (15). We have the trivial solution ψ=±π/2, which corresponds to a shape of constant curvature $\kappa =\pm \omega_{1}$. The energy density for this circular configuration is $E_{0}/L=C\omega_{3}^{2}/2$.

The non-trivial general solution of Equation (14) assuming the condition ψ(0)=0 without loss of generality is well known:

(18) $$\psi \left(s\right)=am\left(s/\lambda |m\right)$$

with a characteristic length $\lambda =\frac{1}{\omega_{1}}\sqrt{\frac{C}{B}m}$. The function am is the Jacobi amplitude function, and m>0. This solution corresponds to the oscillatory and the revolving regimes of the pendulum with m>1 and m<1, respectively. The case m=1 is the homoclinic pendulum (a single revolution of the pendulum).

To each value of the parameter *m* corresponds a different filament shape. However, a helical filament of length *L* with given material parameters will adopt a single ground state when squeezed onto the plane. It can be shown [121] that the value of *m*, which minimizes the elastic energy density E(m)/L of the chain, lies in the interval [0,1]. Shapes with m>1 cannot be the ground state of a squeelix. For m≤1, the minimum of E(m)/L is the solution of the equation [121]:

(19) $$\frac{\sqrt{m}}{E(m)}=\sqrt{\gamma }\;\mathrm{with}:\;\gamma =\frac{4\omega_{1}^{2}B}{\pi^{2}\omega_{3}^{2}C}$$

with E(m) the complete elliptic integral of the second kind. Equation (19) shows that the ground state of a squeelix of infinite length is determined by the parameter *γ*. The left-hand side of Equation (19) is a growing function of *m* that goes from zero to one. Therefore, a minimum of E(m)/L exists for γ≤1 only. When γ>1, the ground state has thus a circular shape.

For γ=1, Equation (19) gives m=1, i.e., the homoclinic pendulum. Equation (18) becomes ψ(s)=2arctan(s/λ)-π/2, which interpolates between ψ(-∞)=-π/2 and ψ(∞)=π/2. We called this configuration a twist-kink [124]. Provided L≫λ as considered here, the twist kink is localized on a distance ∼λ and is separating two curvature flipped regions of constant curvature $\kappa \approx \pm \omega_{1}$. The associated energy is $E_{1}=2\sqrt{BC}\omega_{1}-\pi C\omega_{3}+C\omega_{3}^{2}L/2.$ Therefore, the self-energy of a single twist-kink is:

$$\Delta E=E_{1}-E_{0}=\pi C\omega_{3}\left(\sqrt{\gamma }-1\right)$$

with *γ* the twist-kink expulsion parameter. This terminology speaks for itself as for γ>1, twist-kinks are expelled from the filament. It forms a shape with multiple circles of the same radius $1/\omega_{1}$ on top of each other (see Figure 6).

When γ<1, we have m<1, and ψ(s) is a monotonous function of *s*. The ground state is populated by a finite density of twist-kinks. This density is limited by the mutual kink-kink (with inter-distance *d*) repulsion $U_{\mathrm{int}}∼\pi C\omega_{3}\sqrt{\gamma }f\left(d/\lambda \right)$ where f(x)∼1/x for x≪1 and $f\left(x\right)∼e^{-x}$ for x≫1. This repulsion gets stronger with larger *γ*. For γ≲1, Equation (19) gives m≈γ, which corresponds to a dilute regime with well-separated twist-kinks. In sections of the filament with constant ψ(s)≈±π/2, the curvature is constant too, and the filament locally forms a circular arc with radius $\approx 1/\omega_{1}$. In regions where ψ(s) changes by *π*, curvature reverses its orientation. The reversal points, the twist-kinks, are separating circular arcs of opposite curvature orientation. The filament then consists of a succession of circular arcs (or circles or even spirals, depending on the parameters) with alternating orientations (see Figure 6).

Decreasing *γ* the density of twist-kinks increases until the notion of individual twist-kinks looses its meaning (when d/λ≲1). For γ≪1, Equation (19) becomes $m\approx \frac{\pi^{2}}{4}\gamma$. In this regime, the twist evolves smoothly like ψ(s)∼s. The squeelix changes periodically the orientation of its curvature and has a sinus-like shape (see Figure 6).

Note that for an oscillating pendulum, i.e., m>1, where ψ(s) oscillates periodically, the chain is populated by a finite density of alternating twist-kink and anti-twist-kinks. This oscillatory regime is not the ground state of a squeelix, because the anti-kink configuration has $\psi^{\prime }\left(s\right)<0$ around the inversion curvature point, and this configuration maximizes the torsion contribution to the total energy. These solutions could nevertheless be thermally activated [121].

The theory of squeelices should be useful to determine the material parameters of helical filaments that are often confined in experiments.

#### 4.1.2. Thermally-Injected Twist-Kinks and Hyper Flexibility: The Arc/Arc Toy Model

From the mechanical study of the squeezed helical filament, we found that there is a twist expulsion regime where the ground state is circular. Twist-kinks appear only as thermal excitations, which are penalized by the Boltzmann weight $e^{-E}$ and are localized. We can look at them as quasi-particles. If their separation remains on average much larger than their extension, we can neglect their extension, their mutual interaction and the interaction with the filaments ends. The model boils down to an ideal gas of twist-kinks separating regions of flipped curvature. This toy model we call the arc/arc model [124,125]. The average number of twist kinks is $\langle k\rangle =Ne^{-E}$. As the twist-kinks do not interact their number, *k* is Poisson distributed and $P\left(k\right)=e^{-\langle k\rangle }{\langle k\rangle }^{k}/k!$. In the following, we derive the statistics of the deflection angle for this model. We will account for 2D flexural fluctuations governed by the persistence length *ℓ* and show that these fluctuations factorize out from the partition function in a convolution product. By construction, the toy model misses twist-fluctuations, beyond those associated with the fluctuation of the number and the position of twist-kinks.

To ease the calculations, it is convenient to work in Fourier space for the bending angle and in Laplace space for the arc-length along the filament. In the absence of bending fluctuations the distribution of the increment in angle $\theta -\theta_{0}$ along an arc of length *s* between two twist-kinks is deterministic and obeys $g_{\pm }=\delta \left(\theta -\theta_{0}-\pm \omega s\right)$ for an arc with positive/negative curvature. This can be Fourier transformed with respect to the bending angle increment as $g_{\pm }\left(q,s\right)=e^{\pm iq\omega s}$. Accounting for the Gaussian flexural fluctuations (see Section 1), the angular distribution of the deflection along the arc of length *s* becomes $g_{\pm }\left(q,s\right)e^{-q^{2}s/\ell }$. The deflection angle being additive along the filament, the statistical weight of a given sequence of arcs separated by twist-kinks is the product of the corresponding $g_{\pm }\left(q,s\right)e^{-q^{2}s/\left(2\ell \right)}$ factors with the Boltzmann weights $e^{-E}$ associated with twist-kinks. It is obvious that the Gaussian fluctuation factors multiply to $e^{-q^{2}S/\left(2\ell \right)}$ for any sequence. It is hence enough to calculate the partition sum for infinite stiffness (in its discrete formulation, the problem with infinite stiffness maps on a finite one-dimensional Ising spin system on a segment), without the Gaussian bending fluctuations, which can be taken into account later by a convolution in angular space, as could have been anticipated. We hence just have to calculate the partition sum for the bare $g_{\pm }$ propagators. Note that these partition sums will have a bounded support in the variable *θ*. It is now convenient to get rid of the convolution in the arc length (the arc length adds up to S=Nb) by taking the Laplace transform (in using the Laplace transform, rather than the z-transform, we take a quasi-continuum limit) of the $g_{\pm }\left(q,s\right)$ with respect to n=s/b. This is simply $g_{\pm }\left(q,p\right)=1/\left(p-\pm iq\omega b\right)$ with *p* the Laplace variable conjugated to *n*. The statistical weight of a sequence is now just the product of the associated Fourier–Laplace propagators with the Boltzmann weights of the twist-kinks. Take all configurations starting out and ending with a positive curvature, their partition sum reads $Z_{++}=\sum_{j=0}^{\infty }\;g_{+}\left(q,p\right){\left(g_{-}\left(q,p\right)g_{+}\left(q,p\right)\right)}^{j}e^{-2jE}$. Similar sums $Z_{--}$, $Z_{+-}$ describe other edge curvatures. We may for example evaluate the partition sum *Z* for unbiased starting condition with equal probability 1/2 for either curvature $Z=(Z_{++}+Z_{--}+2Z_{+-})/2$. After some simple algebra:

(20) $$Z\left(q,p\right)=\frac{p+e^{-E}}{p^{2}+q^{2}\omega^{2}b^{2}-e^{-2E}}$$

which yields Z(θ,p) after backwards Fourier transform:

(21) $$Z\left(\theta ,p\right)=\frac{p+e^{-E}}{2\omega b{\left(p^{2}-e^{-2E}\right)}^{1/2}}e^{-\frac{{\left(p^{2}-e^{-2E}\right)}^{1/2}}{\omega b}\left|\theta \right|}$$

which is readily inverted back to *s* space. After normalization to the support $\left[-\omega S,\omega S\right]$, we obtain the even deflection angle distribution function written below for θ>0:

(22) $$\begin{array}{c} P_{\infty }\left(\theta >0,S\right)=\frac{e^{-E}}{2\omega b}e^{-\langle k\rangle }\left\{I_{0}\left(\langle k\rangle \sqrt{1-{\hat{\theta }}^{2}}\right)+\frac{I_{1}\left(\langle k\rangle \sqrt{1-{\hat{\theta }}^{2}}\right)}{\sqrt{1-{\hat{\theta }}^{2}}}\right\}u\left(1-\hat{\theta }\right)+\frac{e^{-\langle k\rangle }}{2}\delta \left(\theta -\omega S\right) \end{array}$$

where $\langle k\rangle =Se^{-E}/b$ and $\hat{\theta }=\theta /S\omega$. The Heaviside function u(x) (defined as u(x)=0 for x<0, u(0)=1/2, u(x)=1 for x>0) was introduced, and the $I_{n}$ are modified Bessel function of the first kind. The last term stands for the ground state contribution, which is exponentially suppressed for large 〈k〉; the other contributions come from excited states and have bounded support as anticipated. To take into account bending fluctuations, it is enough to convolute $P_{\infty }\left(\theta \right)$ with the Gaussian $G\left(\theta \right)\;=e^{-\ell \theta^{2}/\left(2S\right)}/\sqrt{2\pi S/\ell }$,

(23) $$P\left(\theta \right)=P_{\infty }\left(\theta \right)⋆G\left(\theta \right).$$

**Figure 7:**
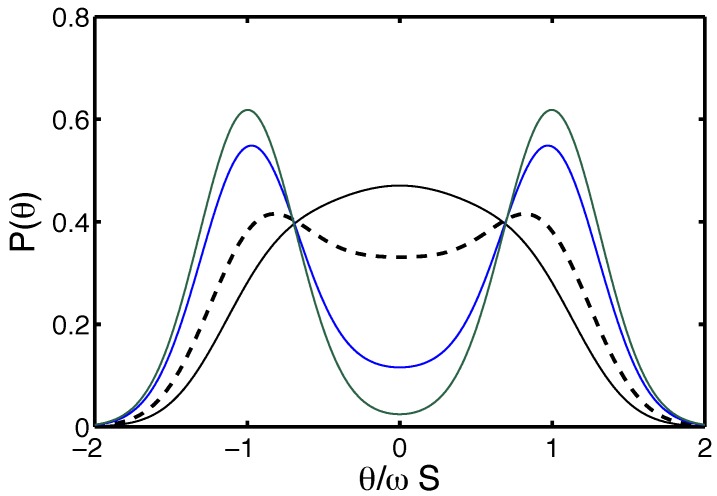
P(θ) for S=100b, ω=0.01/b and ℓ=1000b obtained by convolution according to Equation (23) using Equation (22). The Boltzmann weight of a twist kink is $e^{-E}$. The lines correspond to E= 4 (green), 6 (blue) and 8 (black). The thick dashed line corresponds to E=-log(bω)≈4.6.

When the width of bending fluctuations is small, this merely transforms the delta peak into a Gaussian and smoothens the Heaviside cut-off. It has little effect on the regular part. A series of deflection distributions is shown in Figure 7. Other approaches can be used to derive Equation (21), among those the recursive scheme described in [126], which is a continuous analog of the transfer matrix formulation.

The (few) twist-kinks diffuse rather freely along the chain, which results in augmented shape fluctuations (also reflected by the distribution P(θ)) and hyper-flexibility, despite the intrinsic flexural rigidity. The mean square end-to-end distance $R_{e}^{2}$ and the radius of gyration $R_{g}$ are measurable quantities and interesting on their own. As noticed below, Equation (1), they can be derived from the angular correlation C(s), which is given by the (real part) of the Fourier transformed normalized partition function Z(q) at q=1:

(24) $$\begin{array}{ccc} C(s) & = & \left(\frac{1}{2}-\frac{1}{2\sqrt{1-\omega^{2}b^{2}e^{2E}}}\right)e^{-s\left[\frac{e^{-E}}{b}\left(1+\sqrt{1-\omega^{2}b^{2}e^{2E}}\right)+\frac{1}{2\ell }\right]}\\ & & +\left(\frac{1}{2}+\frac{1}{2\sqrt{1-\omega^{2}b^{2}e^{2E}}}\right)e^{-s\left[\frac{e^{-E}}{b}\left(1-\sqrt{1-\omega^{2}b^{2}e^{2E}}\right)+\frac{1}{2\ell }\right]} \end{array}$$

The correlation C(s) is oscillatory if one turn is not destroyed by twist-kinks ($\omega be^{E}>1$) and decays monotonically otherwise. As $R^{2}$ and $R_{g}^{2}$ are linear functionals of C(s), they can be written as combinations of similar quantities for the WLC given in Equation (2):

(25) $$R_{e,g}^{2}=\frac{1}{2}\left[R_{e,g}^{2}\left(\ell_{1}\right)+R_{e,g}^{2}\left(\ell_{2}\right)\right]+\frac{1}{2\sqrt{1-\omega^{2}b^{2}e^{2E}}}\left[R_{e,g}^{2}\left(\ell_{1}\right)-R_{e,g}^{2}\left(\ell_{2}\right)\right]$$

with $1/\left(2\ell_{1}\right)=\frac{e^{-E}}{b}\left(1-\sqrt{1-\omega^{2}b^{2}e^{2E}}\right)+\frac{1}{2\ell }$ and $1/\left(2\ell_{2}\right)=\frac{e^{-E}}{b}\left(1+\sqrt{1-\omega^{2}b^{2}e^{2E}}\right)+\frac{1}{2\ell }$. The parameters $\ell_{1,2}$ are complex conjugate in the oscillatory C(s) regime, so are the associated $R_{e,g}^{2}$, which are hence not physical. However, the $R_{e,g}^{2}$ for the helical filament as given by Equation (25) are real.

**Figure 8:**
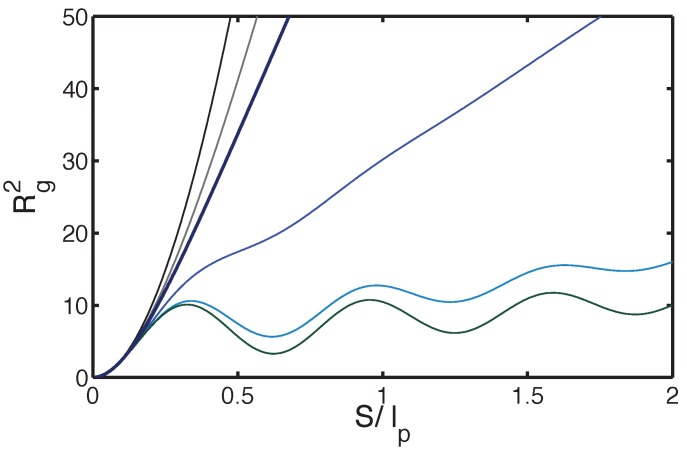
$R_{g}^{2}\left(S\right)$ for ω=0.01/b and $l_{p}=1000b$. The Boltzmann weight of a twist kink is $e^{-E}$. The thin lines are for E=2,4,6,8 and 10 from top to bottom. The thick line indicate $R_{g}^{2}$ with E=-log(bω)≈4.6. Undulations appear for E>-log(bω).

Figure 8 shows the squared radius of gyration as a function of the contour length *S* for a typical set of parameters. As suggested by Equation (25), for long enough chains, there is a flexible regime with an effective average persistence length. In the linear response regime of vanishingly weak force, the fluctuation $R_{e}^{2}$ also gives the response function, and the average elongation *x* along the direction of the force *f* reads $\langle x\rangle =\frac{R_{e}^{2}}{2k_{B}T}f$. As was anticipated from mechanics, a somewhat larger force, typically in the pN range, affects the distribution of twist-kinks, entailing a non-linear regime. Below, we present the free energy map as a function of the twist angle *ψ* (given in units of *π*) and the extension *D*. We first give the map without applied force for the full Hamiltonian of the squeezed helical filament and show how it deforms under an applied force.

#### 4.1.3. The Free Energy Map

**Figure 9:**
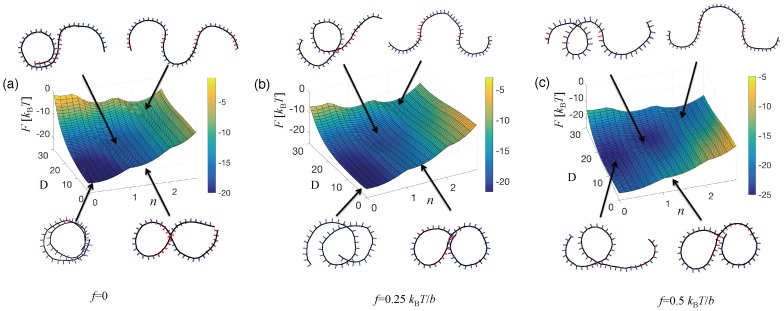
(**a**) Free energy landscapes without applied force (f=0) and (**b, c**) under increasing force ($f=0.25k_{B}T/b$, $f=0.50k_{B}T/b$). To fix the force scale: for b=0.5 nm, given $k_{B}T\approx 4$ pNnm, $k_{B}T/b\approx 8$ pN.

The free energy map F(ψ,D) keeping track of the extension *D* and of the number of twist-kinks *n* related to the global twist *ψ* by n=ψ/π, is obtained from a Wang–Landau Monte Carlo simulation [124,127]. The parameters appearing in the full Hamiltonian take the following values: $\omega_{1}=0.26$ b${}^{-1}$, $\omega_{3}=0.1$ b${}^{-1}$, which correspond to the twist-expulsion parameter γ=5.47. The chain length is rather short N=48, and edge effects are present. The helical filament is squeezed to the vicinity of the plane z=0 by a harmonic potential of rigidity $25k_{B}T/b^{2}$. Under an external force f, the Gibbs free energy *G* is obtained as $G=F-k_{B}T\log\left(I_{0}\left(fD\right)\right)$, where the relative orientation of the end-to-end vector and the force is integrated out and $I_{0}\left(x\right)$ stands for the modified Bessel function. At large force, the end-to-end vector merely aligns with the force and the log simplifies to ≈fD; at small force, all orientations contribute, and we recover the linear response result. Figure 9 shows how a force destabilizes the ground state, which corresponds to a two-turn circular shape. The vicinity of excited state n=1 gets more populated under a force, and the associated *D* is better defined. This documents the injection of twist-kinks and a better localization of a single twist-kink under the applied force. It is easy to get a good intuition of these phenomena from the arc/arc model.

### 4.2. Emergence of Bistability

#### 4.2.1. Long Range Elasticity and Switchability

Nature is known to play many variations on its basic mechanical components. With semiflexible chains being so abundant in living organisms, it is no surprise that they come with many interesting extras and tweaks that often serve to fine-tune their function. Two of these anomalous filament characteristics are bistability and shape cooperativity. How and when do these features emerge?

**Figure 10:**
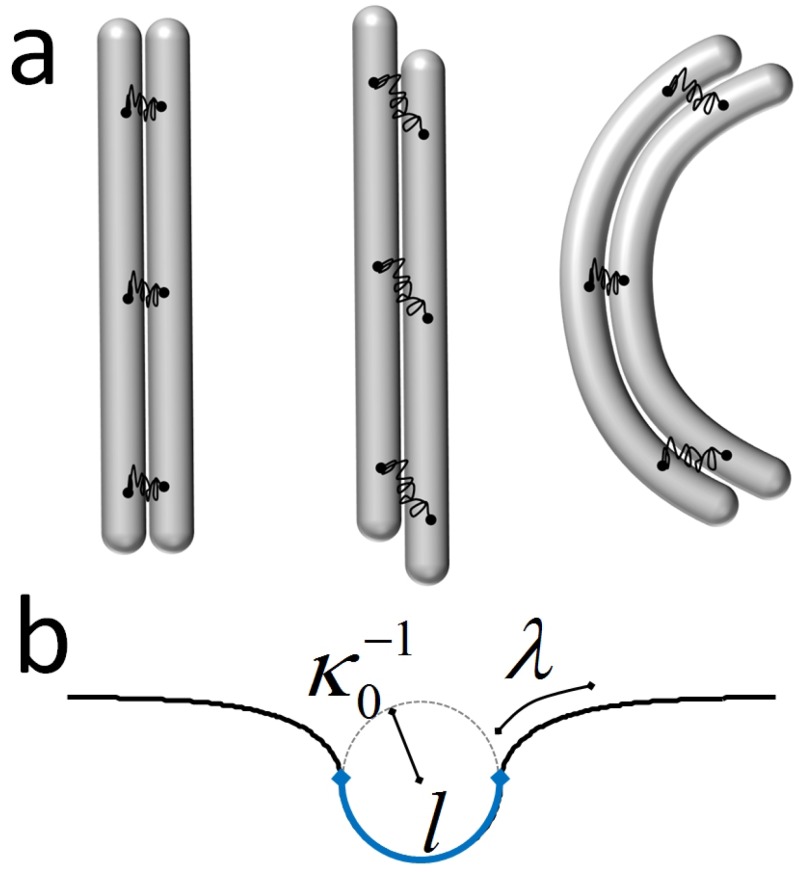
(**a**) Two filaments are coupled with elastic springs forming a simple two-chain bundle; (**b**) the shear and bending degrees of freedom become strongly coupled, leading to long-range deformation effects. An arc formed in the region of length *l* (blue line) around the origin induces two counter-arcs with opposite curvature in its next proximity. The deformations are screened and vanish only at length scales longer than the elastic screening length *λ*.

**Figure 11:**
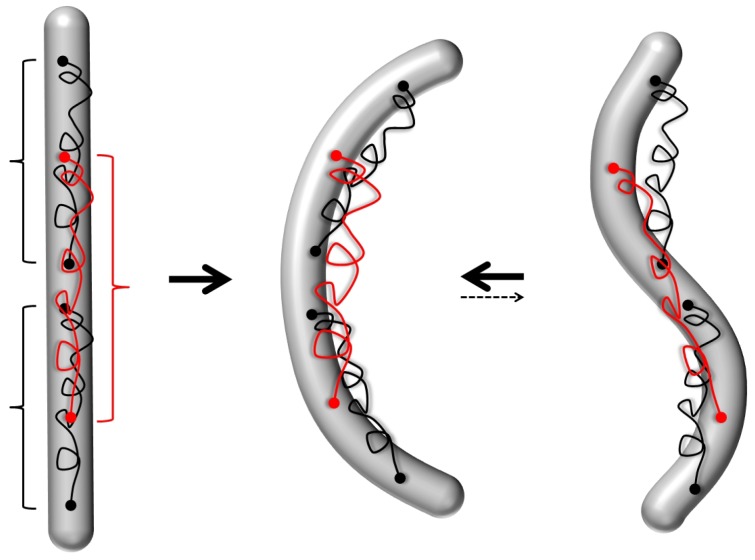
A semiflexible filament with elastic tails that cross-link points along its backbone becomes bistable. If the cross-linking point intervals overlap in addition (red and black chains), the curvature switching becomes cooperative. The single arc state in the middle is the energetically most stable (indicated by bold and dashed arrows).

To answer this question, note that biofilaments (and semiflexible chains in general) are intrinsically prone to simple modifications that generate a long range shape coherence along the filament contour and/or destroy their unique straight ground state, cf. Figure 10 and Figure 11. As an example, consider a simple cross-linking of two semiflexible chains by soft spring connections (like, e.g., flexible polymers). This object behaves radically differently from a WLC on short and intermediate scales. In addition to the bending energy per length $∼B\theta^{\prime 2},$ with $\theta \left(s\right)$ the tangent angle at position *s* and Btwice the bending constant of a single chain, there is also an inter-chain shear deformation $\tau \left(s\right).$ The latter gives a second elastic contribution, the shear energy $∼K\tau^{2}.$ The elastic shear constant is given by $K∼\rho_{ch}w^{2}k\;$ for this simple (two filament) bundle of typical lateral width w, the elastic spring constants *k* of the individual cross-connecting chains with chain line density $\rho_{ch}$,

(26) $$E=\frac{1}{2}\int B\theta^{\prime 2}\left(s\right)+K\tau^{2}\left(s\right)ds$$

In the limit where the axial stretching of the two filaments can be neglected, the shear deformation at any given location *s* along the contour can be expressed in terms of *θ*:

(27) $$\tau \left(s\right)=\theta \left(s\right)-\bar{\theta }$$

with the average angular orientation:

$$\bar{\theta }=L^{-1}\int \theta \left(s\right)ds.$$

The energy (26) with the constraint (27) can be interpreted (for weak angular deformations θ≪1) as a WLC under strong tension. This apparent tension is peculiar as it is not applied from the outside, but rather behaves as an internal “self-tension” that acts with respect to the mean internal orientation $\bar{\theta }$. This gives rise to long-range curvature-curvature interactions. For instance, it is easy to see that if a fixed curvature $\kappa_{0}$ is imposed within a certain region of length *l* around some position $s_{0}$, it will automatically induce opposite curvature in its proximal regions, cf. Figure 10b. Far away from $s_{0}$, the shear and bending deformations decay exponentially:

$$\theta^{\prime }\propto \tau ∼\kappa_{0}l\exp\left(-|s-s_{0}|/l\right)$$

with a characteristic screening length scale set by:

$$\lambda =\sqrt{B/K}.$$

The elastic screening effect is a notable characteristic of such a model. If the preformed arc is short, l≪λ, its total energy:

$$E_{\mathrm{arc}}∼\sqrt{BK}\kappa_{0}^{2}l^{2}$$

scales quadratically with its length, very much unlike the classical WLC model, where $E_{\mathrm{arc}}\propto l$. For longer fixed curvature arcs, the shear energy dominates over bending and grows even quicker with $E_{\mathrm{arc}}∼K\kappa_{0}^{2}l^{3}.$ Each piece of the arc interacts with any other one in a non-local manner, and it becomes increasingly costly to form longer arcs (the energy density $E_{\mathrm{arc}}/l$ grows in a size-dependent manner). In the non-stretchable filament limit, this model was dubbed “the railway track model” by Everaers et al. [128]. More elaborate, but in spirit similar versions of it, called the “soft shear” models, have gained some attention in describing long-range deformation modes and correlated reshaping of microtubules [129,130,131]. The main idea there is that the microtubule’s subunits, the tubulin protofilaments, are very rigid filamentous entities, coupled by very soft lateral bonds, like in the railway-track model. Although an attractive theoretical idea, no structural biology data so far support such a large anisotropy. Despite that, there is mounting evidence for some longer range interaction along the microtubule lattice [132]. However, based on current experimental evidence, the nature of microtubule long-range curvature deformations appears to be more likely positive cooperative, rather than anti-cooperative, as the railway track model and its derivatives imply. This calls for more radical revisions of the WLC model.

#### 4.2.2. Bistability and Cooperativity

Real biological filaments have many internal degrees of freedom even at the level of a single monomer unit or the interface of two of them. For instance, DNA bases can flip, tilt and interact with the orientation of the sugar phosphate backbone, giving rise to discrete A, B and Z forms of DNA [133,134]. The microtubule’s elementary units (tubulin dimers) act as curvature switchable elements [132], and actin filaments can switch their inter-unit twist [135]. Many other examples of biofilament multi-stability emerge due to the monomer’s molecular complexity. However, even generic interactions along the backbone-like tail bridging or geometrical constraints, including confinement to surfaces, can break the symmetry and uniqueness of the filament’s ground state. In many cases, multi-stability cannot be simply averaged out on biologically-relevant scales, especially when positive cooperativity between the unit conformations comes into play.

For illustration, reconsider the previous case of a squeezed 3D helix that is forced to a 2D surface. By forcing it onto a surface, its elastic degrees of freedom (bending and twisting) become mutually strongly coupled. The induced non-linearities lead to a symmetry breaking of the ground state. The repulsion of the torsional twist kinks, which coincide with the curvature switching regions of such chains, induces an effective cooperative interaction between the neighboring curved monomers. This effectively leads to large blocks of monomers, which form positive and negative bistable curvature regions.

Of course, in this example, one might rightly object that the monomers of the chain when seen in their own material reference frame do not really switch. Only the projected signed curvature (of the centerline) can assume two values, which from the perspective of the monomers only amounts to a rotation of the monomers around the filament axis.

Another generic example for a prestress-induced bistability is a semiflexible biofilament with elastic tails that span along the filament’s backbone and pairwise connect distant points at typical distance *d* with spring constants k, cf. Figure 11. When the filament is straight, the springs are in their extended (prestressed) state with length *d* and and have an energy $E_{ch}∼\frac{1}{2}kd^{2}$. In the following, let us consider only the simplest planar filament case. The shape of such a 2D “condensed tail WLC” is determined by a competition of the stretching energy of the flexible chains and the bending energy of the semiflexible filament. The chains have the tendency to buckle the filament and form positive/negative curvature buckles of the typical size *d* once:

(28) $$k>k_{\mathrm{crit}}=12Bd^{-3}.$$

Close to the buckling transition $k⪆k_{\mathrm{crit}}$ and on length scales ≫d, the energy can be expanded and is (up to a constant):

(29) $$E_{b}\approx \frac{kd^{3}}{2}\int \left[C_{1}\left(\frac{k_{\mathrm{crit}}}{k}-1\right)\kappa^{2}+C_{2}d^{2}\kappa^{4}\right]ds$$

with $C_{1}$,$C_{2}>0$ numeric constants. Such a system is bistable and displays curved sections with switchable curvature:

(30) $$\kappa \propto \left(\pm \right)\frac{\sqrt{1-k_{\mathrm{crit}}/k}}{d}.$$

If the chains attachment intervals do not overlap, the curvature switching is local and non-cooperative on distances larger than *d*. However, in the more generic case when they overlap (cf. red chains in Figure 11), there is an additional cooperative coupling term emerging:

(31) $$E_{\mathrm{couple}}\approx \frac{H}{2}\int \kappa^{\prime 2}ds$$

with $H\propto kd^{5}$ a higher order “hyper-stiffness” constant. Note that the latter gives rise to a persistence of curvature, rather than the usual persistence of tangent angle, as for the WLC.

This toy model is less academic than one would think. Classical biofilaments, like microtubules, have naturally built-in long amorphous polymer tails (one per monomer) that can span in their stretched state to nearest neighbors or beyond. Furthermore, actin filaments and microtubules often interact with other polymer tail-forming proteins that come as an integral part of the cytoskeleton (including tau proteins, various MAPs, formins). Switchability, in particular of microtubules, might partially relate to these tails.

Whatever its origin, there has been mounting experimental evidence, for the bistability of the microtubule lattice [132]. The next section (4.3) is devoted to microtubules.

**Figure 12:**
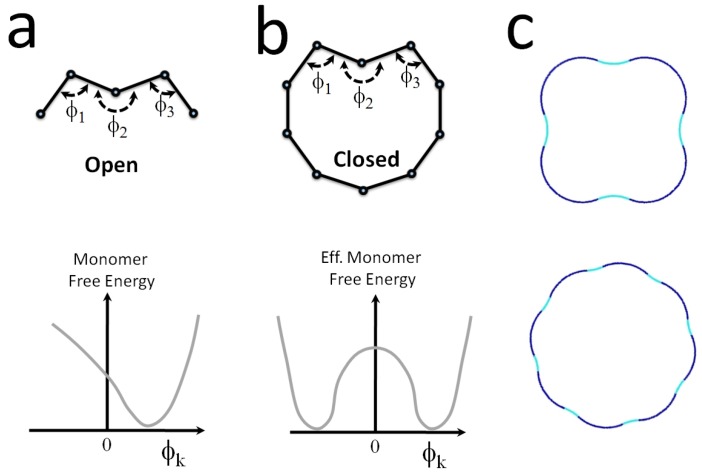
Polymorphic crunching: (**a**) Nonlinear bendable units are coupled in-plane of bending by a ring closure constraint (**b**). The constraint modifies their effective free energy and gives rise to a bistable monomer potential. (**c**) Two out of exponentially many energetically-equivalent ground state realizations. Blue and light-blue indicate the regions of opposite curvature.

Another way for the monomer to become bistable and attain long-range interactions has been discussed in [136], where units with a non-linear angular potential are constrained into circular geometry, cf. Figure 12. What happens when protein monomers with an intrinsic curvature form a stiff polymer, which is forced to close in a ring of a different radius of curvature?

When the ring curvature differs from the preferred curvature of each unit, the monomers transform from mono-stable into fluctuating bistable units. This has rather dramatic effects on the overall shape of the ring. In particular, the ground state becomes highly degenerate, with an exponentially-growing number of realizations with the number of monomers *N*. When the filament thermally explores this exponentially-degenerate energy landscape, it exhibits anomalous fluctuations giving rise to an effectively reduced length-dependent persistence length. That can be interpreted as a paradoxical effective softening of the filament despite the high frustration in the form of mechanical prestress, which would naively give rise to a hardening in the system. Another interesting theme in this example is that the global integral constraint on the local curvatures $\kappa \left(s\right)$, $\int \kappa \left(s\right)ds=2\pi ,$ not only induces bistability, but also gives rise to a long range, whole system-spanning conformational interaction between the units. Close to the ground state, switching of one unit’s angle to one state forces another unit, anywhere along the ring to switch to the opposite state to satisfy for the global constraint. In a continuous representation, the degeneracy is lifted by a penalty $\propto \kappa^{\prime 2}$ (see Equation (31)) or by the domain wall energy between blocks of different curvature in a discrete model.

### 4.3. Polymorphic Model of Microtubules

There are numerous indications for microtubules having properties beyond the WLC model. Examples occurring in vivo are their curling-up , with radii of curvature of the order of micron, during the formation of synapses [110,111] and during blood platelet formation by megakaryocytes [137]. In vitro, similar curling-up events are found in kinesin-gliding assays, i.e., when microtubules are transported along a molecular motor-covered substrate [112,113].

**Figure 13:**
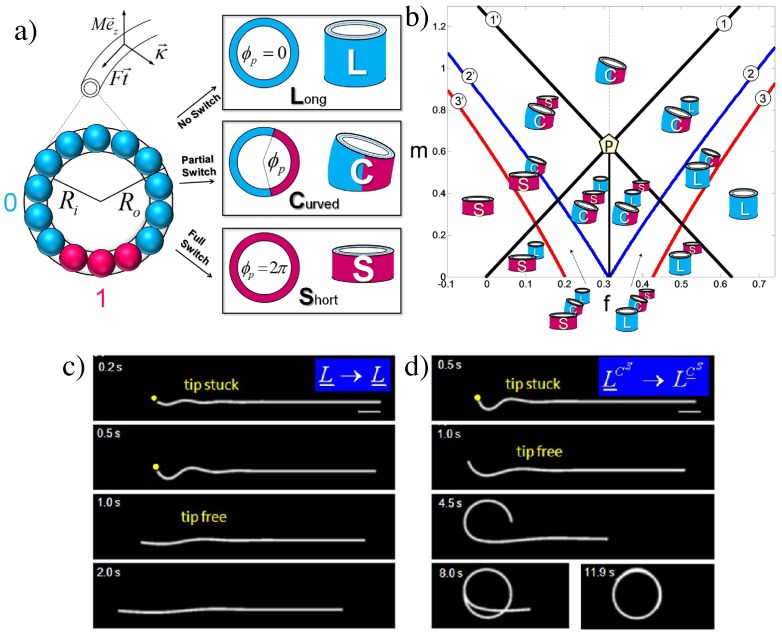
The polymorphic model of microtubules. (**a**) The switchability of tubulin dimers leads to a competition of three states of the microtubule’s cross-section: straight and long (L), curved (C) and straight and short (S). (**b**) “Phase diagram” of the polymorphic microtubule model as a function of generalized force *f* vs. torque *m*. The existing states in the respective regions are ordered by their polymorphic energies, the one at the bottom having minimum energy. (**c**) An intermittent buckling event, for a microtubule transported along a motor-covered surface, in case of f≃0.7 (corresponding to small switching energies ΔG≃0). The behavior observed is the one of a regular WLC chain. (**d**) An intermittent buckling event in case of f≃0.4 (corresponding $\Delta G≃5\;k_{B}T)$. The microtubule curls up.

These observations can be rationalized by taking aspects of the microtubules’ microscopic structure into account, namely the possibility of conformational changes of their subunits, the tubulin dimers. The hollow tube structure of the microtubules is formed by 9–15, typically 13, protofilaments, which, in turn, are formed by head-to-tail polymerization of the tubulin dimers. Experiments on isolated, taxol-stabilized protofilaments have shown their propensity of being curved [138,139], indicating that curved conformations are energetically preferred. The distribution is bimodal, with radii of curvature predominantly at 20–30 nm (“strongly-curved” state), and at 250 nm (“weakly-curved state”). A second important piece of information, obtained earlier by electron cryo-microscopy, is that the microtubule lattice displays two elongational states [140], one of them being about 2% shorter than the other. Both facts can be brought in harmony by assuming that the curved conformations of the dimers are slightly shorter (cf. also Figure 13a).

The polymorphic model of microtubules [141] incorporates these facts as follows, cf. Figure 13: it assumes that a tubulin dimer can reduce its free energy by |ΔG| by switching to the curved state. In turn, the curved state is accompanied by elastic strains. The tubulins in the cross-section, marked as spheres in Figure 13a, are treated as a two-layered structure. If the tubulin is in the straight conformation (blue in Figure 13a), there is no preferred strain. However, in the curved conformation (purple in Figure 13a), the inner part (for radius $\rho \in [R_{i},R_{m}]$) and the outer one (for $\rho \in [R_{m},R_{o}]$; with $R_{i},R_{o}$ the inner and outer radius of the microtubule), the respective tubulin subunit experiences preferred strains $\varepsilon_{i}>0$ (tensile) and $\varepsilon_{o}<0$ (compressive) in the inner and outer layer, respectively, reflecting preferred outwards curving. This simple model can explain the curling-up as follows: when the curved protofilaments are constrained in the microtubule’s lattice, they cannot all bend as they would intrinsically prefer; the microtubule is a frustrated, mechanically prestressed system. Nevertheless, states where few neighboring protofilaments are curved, and hence the whole microtubule, while the others have to accommodate and pay a bending energy penalty, can indeed have lower energy than the completely straight state.

This conjecture can be quantified by calculating the total energy of a cross-section, having two parts: first, the switching energy (with n∈[1,N] the index of each tubulin with e.g., N=13):

(32) $$e_{\mathrm{switch}}=\frac{\Delta G}{b}\sum_{n}\sigma_{n}$$

with b≈8nm the dimer size and $\sigma_{n}=0$ ($\sigma_{n}=1$) for the straight (curved) conformation, respectively. Second, the elastic energy:

(33) $$e_{\mathrm{el}}=\frac{Y}{2}\int_{R_{i}}^{R_{o}}\rho d\rho \int_{0}^{2\pi }d\phi {\left[\varepsilon \left(\rho ,\phi \right)-\varepsilon_{\mathrm{pref}}\left(\rho ,\phi \right)\right]}^{2}\;,$$

where $\varepsilon \left(\rho ,\phi \right)=-\vec{\kappa }\cdot \vec{\rho }+\bar{\varepsilon }$ is the inner strain (with $\vec{\kappa }=\left(\kappa_{x},\kappa_{y}\right)$ the centerline curvature, $\bar{\varepsilon }$ the mean stretching strain) and $\varepsilon_{\mathrm{pref}}$ the preferred strain discussed above (i.e., $\varepsilon_{\mathrm{pref}}=0$ if the respective tubulin at *φ* is straight, and $\varepsilon_{\mathrm{pref}}=\varepsilon_{i/o}$ in the inner/outer layers when it is curved).

This energy has been studied extensively in [141]. Since switched dimers effectively strongly attract each other within the lattice, one can consider all switched dimers to form a single block. The angular size *p* of the switched block, in turn, defines the “polymorphic variable” (or order parameter):

(34) $$\phi_{p}=2\pi \frac{p}{N}\;:$$

for $\phi_{p}=0$, all tubulins and, hence, the microtubule are straight. In case $\phi_{p}=2\pi$, all tubulins are switched to curved; hence, the microtubule is also straight, but shortened due to the intrinsic strains. In case $\phi_{p}$ is in between these values, the microtubule is curved, cf. Figure 13a.

Using this new variable, it is straightforward to evaluate $e_{\mathrm{tot}}=e_{\mathrm{el}}+e_{\mathrm{switch}}+F\bar{\varepsilon }+M\kappa$, including an externally-applied force *F* and torque *M*. In doing so, a characteristic curvature can be identified, which reads:

(35) $$\kappa_{1}=\frac{8}{3\pi }\frac{\varepsilon_{i}\left(R_{m}^{3}-R_{i}^{3}\right)+\varepsilon_{o}\left(R_{o}^{3}-R_{m}^{3}\right)}{R_{o}^{4}-R_{i}^{4}}\;.$$

Minimizing the total energy with respect to strain ($\bar{\varepsilon }$) and curvature (*κ*), assuming that these adjust to the external loads, yields the “polymorphic potential”:

(36) $$e_{\mathrm{tot}}\left(\phi_{p}\right)=B\kappa_{1}^{2}\left[-\frac{c_{p}}{2}\phi_{p}^{2}+f\phi_{p}-m\sin\frac{\phi_{p}}{2}-\frac{1}{2}\sin^{2}\frac{\phi_{p}}{2}\right]\;,$$

as a function of the rescaled, generalized force *f* and torque *m*. The generalized force contains the external force, a contribution from the lattice constraints and most importantly a contribution linear in the switching energy ∝ΔG.

Figure 13b shows that for appropriate loading situations (given *m* and *f*), states with $\phi_{p}\neq 0,2\pi$ can have minimum energy. The microtubule will hence be macroscopically curved, with a curvature close to the characteristic one, $\kappa_{1}$. Importantly, using the measured curvature of protofilaments (in the highly curved state) and the 2% shortening of the microtubule, the preferred strains can be determined, and together with the known inner and outer radius of the microtubule, Equation (35) can be evaluated to yield $\kappa_{1}≃{\left(1\;\mu m\right)}^{-1}$, as observed experimentally (for the weakly-curved protofilament state, one gets $\kappa_{1,\mathrm{weak}}≃{\left(10\;\mu m\right)}^{-1}$ [142]).

To exemplify the polymorphic microtubule dynamics, [141] considered microtubules gliding on motor carpets, getting temporarily stuck and then continue to move on. It could be shown that such intermittent buckling events, under forces in the order of 10 pN, can easily lead to complete curl-up of microtubules, cf. Figure 13d), if the switching energy is about $\Delta G≃5\pm 1.5\;k_{B}T$. Interestingly, such buckling events correspond to hysteretic paths in the m-f plane of the energy, Equation (36). The finally obtained, curled-up state is only metastable (i.e., a local minimum). In fact, also experimentally, curled-up microtubules are stable only for several 10s of seconds. In case the switching energy is low, the classical WLC behavior is regained; see Figure 13c).

## 5. Miscellaneous Topics and Outlook

Despite its long history, the WLC model is not yet completely explored. We only addressed some aspects of WLC at interfaces; more are listed below. Even in this classical corpus, there remain rather subtle issues often linked to excluded volume interactions in denser systems. The WLC model was very successful to describe stretched d-stranded DNA in its B-phase. This made the model very popular in biophysics, where it tends to be used for all kinds of biofilaments. There is increasing evidence that the mechanics and dynamics of biofilaments are not entirely captured by the WLC model. We discussed several models, which go beyond the WLC. Dedicated experiments, like the study of doubly-confined actin or vimentin filaments in microfluidic channels [143], computer simulations [124] and analytical theory [124,141,142] are all very recent. Understanding the physics of biofilaments in relation with their biological function appears as a major challenge.

Many important issues on semiflexible chains at interfaces, even some related to work in our group, could not be addressed. We list a few and refer the reader to published work, whenever possible.

- We restricted our study to adsorption from dilute solution and mentioned that nematic order is expected in very dense solutions or melts. Even if the nematic order is not thermodynamically stable in the bulk, there may be a thin layer of thickness *ℓ* closest to the wall where orientational order prevails as described in work by Milner [144]. Strong adsorption of WLC in the melt has been simulated recently [145] with a focus on the distribution of loops and tails, layering effects closest to the wall and local mobility.
- We only considered adsorption on undeformable planar surfaces. Biofilaments can deform soft surfaces like membranes to optimize their surface binding [146]; for example, when the orientation for adsorption is not compatible with the in-plane orientation of the preferred curvature. WLCs have to adapt to the curvature of undeformable shells to adsorb, and this can lead to special arrangements as found in [147]. Surfaces bearing fixed obstacles alter the 2D dynamics of a WLC [148,149].
- In this paper, we focused on WLC adsorption. A related issue is forced desorption under an applied tension [150]. This was also analyzed in the context of Hyston/d-DNA complexes [151,152].
- Another way to fix polymers on a surface is end-grafting, earlier mentioned in the latest stage of irreversible adsorption. One recent simulation work compares single end graft and middle graft WLC in great detail [153], which is related to the distributions of loops and tails discussed above (Section 3.1). The role of stiffness in densely-grafted brushes was considered early by Pickett and Witten [154]. More recently, simulations in the Binder group discuss grafted WLC in the brush regime and the crossover to the dilute surface regime (so-called mushrooms) [155].
- Anisotropic filaments (tapes) with two flexural moduli [156,157] do occur; examples are synthetic ladder polymers or short (rigid) associated polypeptides. Helical polypeptide tapes [158] can reassemble in a hierarchy of structures. Similar associations play a role in amyloid diseases [158]. Mesini and coworkers studied a family of organogelators and report various self-assembled structures, including microtubes [159]. Recently, the adsorption of helical tapes on rigid surfaces was considered by Quint [160].
- It is sometimes argued that the reptation tube can be represented by some transverse (in a first approximation harmonic) potential [161]. The motion of a WLC confined to an effective tube by a transverse harmonic potential was considered by several authors [21,162]. The loosely-related topic of polymers in random media was considered in [163].
- Some larger scale man-made chains can be considered as semiflexible. The mechanics of semiflexible chains formed by poly(ethylene glycol)-linked paramagnetic particles was studied by Gast and coworkers [164] as a function of the length of the poly(ethylene glycol) spacer.
- Concerning the helical filaments squeezed in 2D discussed in Section 4.1, many open questions remain in the specific biological contexts. Open questions pertain also to the nonequilibrium behavior of squeelices: e.g., when they are transported along molecular motor-covered substrates, where they display circular, spiraling or wavy trajectories [165] that are also observed experimentally [112,166].
- The investigation of cooperativity and switchability discussed in Section 4.2 and Section 4.3 has only just began. For instance, there are indications for cooperative binding of molecular motors on microtubules [167], for which cooperative conformational changes in the tubulins may be responsible [131]. The polymorphic switching model discussed in Section 4.3 has so far only been employed to taxol-stabilized microtubules, for which there is the most experimental evidence (as taxol is the main microtubule stabilizer preventing further polymerization/depolymerization). In fact, many other molecules, especially microtubule-associated proteins, may also induce conformational changes of tubulin upon binding with different associated energy gains ΔG. This subject deserves further study as a new route for microtubule regulation that goes beyond the regulation of spatial distribution and length (as typically discussed in biology) towards a regulation of their mechanical behavior and response.

## Figures and Tables

**Table 1:** Monomer size *b* along the backbone and persistence length *ℓ* for various polymers (in water if not specified otherwise) assuming worm-like-chain (WLC) statistics: polyethylene (PE) in dodecanol1, polyisobutylene (PIB) in benzene, polydimethylsiloxane (PDMS) in hexane, atactic polystyrene (PS) in cyclohexane, poly(sodium styrene sulfonate) (NaPSS), poly(diallyl-dimethyl ammonium chloride) (PDADMAC), hyaluronan (HA), duplex-DNA (d-DNA), intermediate filament vimentin (IF) and F-actin. The polymers marked by ⋆ are polyelectrolytes measured in water at high salt; for HA, two sets of values for *ℓ* are found in the literature possibly linked to association phenomena. The last three polymers are biofilaments measured in physiological conditions. It is questionable whether the simple WLC model is directly applicable to them (see the last section), and the persistence length may be only indicative.

	*ℓ* (nm)	*b* (nm)	ℓ/b
PE (Dodecanol1)	0.59	0.13	4.5
PIB (Benzene)	0.59	0.26	2.3
PS (Cyclohexane)	0.86	0.26	3.3
PDMS (Hexane)	0.57	0.29	2
${}^{⋆}$NaPSS (high salt)	1.0	0.25	4
${}^{⋆}$PDADMAC (high salt)	3.0	0.47	5.3
${}^{⋆}$HA (high salt)	4–5; 7–10	1.0	4–5; 7–10
d-DNA	50	0.34	150
IF	10${}^{3}$	∼10	∼100
F-actin	17 × 10${}^{3}$	5	3400

**Table 2:** Characteristic length scales for chemisorption of a single worm-like chain. The typical zipping loop size is $s_{0}$. For long chains $S>S^{⋆}$, zipping occurs from multiple nucleation points distant along the chain.

	*ℓ* (nm)	*b* (nm)	$s_{o}$ (nm)	$S^{⋆}$ (nm)
PE (dodecanol1)	0.59	0.13	0.21	1.6
PIB (benzene)	0.59	0.26	0.34	1
PS (cyclohexane)	0.86	0.26	0.38	1.9
PDMS (hexane)	0.57	0.29	0.36	0.90
NAPSS (high salt)	1.0	0.25	0.39	2.5
PDADMAC (high salt)	3.0	0.47	0.80	7.6
HA (high salt)	4–5; 7–10	1.0	1.6–2	12–35
d-DNA	50	0.34	∼1.8	∼ 1.4 × 10${}^{3}$
IF	10${}^{3}$	∼ 10	∼46	∼ 2 × 10${}^{4}$
F-actin	17 × 10${}^{3}$	5	∼75	3 × 10${}^{6}$
